# G2/M checkpoint regulation and apoptosis facilitate the nuclear egress of parvoviral capsids

**DOI:** 10.3389/fcell.2022.1070599

**Published:** 2022-12-08

**Authors:** Salla Mattola, Elina Mäntylä, Vesa Aho, Sami Salminen, Simon Leclerc, Mikko Oittinen, Kari Salokas, Jani Järvensivu, Satu Hakanen, Teemu O Ihalainen, Keijo Viiri, Maija Vihinen-Ranta

**Affiliations:** ^1^ Department of Biological and Environmental Science and Nanoscience Center, University of Jyvaskyla, Jyvaskyla, Finland; ^2^ BioMediTech, Faculty of Medicine and Health Technology, Tampere University, Tampere, Finland; ^3^ Celiac Disease Research Center, Faculty of Medicine and Health Technology, Tampere University Hospital, Tampere, Finland; ^4^ Institute of Biotechnology and Helsinki Institute of Life Science (HiLIFE), University of Helsinki, Helsinki, Finland

**Keywords:** canine parvovirus, nuclear egress of capsids, CRM1, G2/M checkpoint, cyclin B1, apoptosis

## Abstract

The nuclear export factor CRM1-mediated pathway is known to be important for the nuclear egress of progeny parvovirus capsids in the host cells with virus-mediated cell cycle arrest at G2/M. However, it is still unclear whether this is the only pathway by which capsids exit the nucleus. Our studies show that the nuclear egress of DNA-containing full canine parvovirus. capsids was reduced but not fully inhibited when CRM1-mediated nuclear export was prevented by leptomycin B. This suggests that canine parvovirus capsids might use additional routes for nuclear escape. This hypothesis was further supported by our findings that nuclear envelope (NE) permeability was increased at the late stages of infection. Inhibitors of cell cycle regulatory protein cyclin-dependent kinase 1 (Cdk1) and pro-apoptotic caspase 3 prevented the NE leakage. The change in NE permeability could be explained by the regulation of the G2/M checkpoint which is accompanied by early mitotic and apoptotic events. The model of G2/M checkpoint activation was supported by infection-induced nuclear accumulation of cyclin B1 and Cdk1. Both NE permeability and nuclear egress of capsids were reduced by the inhibition of Cdk1. Additional proof of checkpoint function regulation and promotion of apoptotic events was the nucleocytoplasmic redistribution of nuclear transport factors, importins, and Ran, in late infection. Consistent with our findings, post-translational histone acetylation that promotes the regulation of several genes related to cell cycle transition and arrest was detected. In conclusion, the model we propose implies that parvoviral capsid egress partially depends on infection-induced G2/M checkpoint regulation involving early mitotic and apoptotic events.

## Introduction

At the late stages of parvovirus infection, the nuclear assembly of progeny capsids is accompanied by virus-induced cell cycle arrest at G2/M (Adeyemi et al., 2010; Ruiz et al., 2011). Viral activities also stimulate apoptosis (Ran et al., 1999; Poole et al., 2004; Chen et al., 2010; Hristov et al., 2010; Nykky et al., 2010; Mincberg et al., 2011). Although these processes are known to be essential for the progress of the infection, it is not clear how the regulation of these processes is connected to the prelytic nuclear egress of progeny capsids.

Parvoviruses are small non-enveloped DNA viruses with a linear single-stranded DNA genome and only a very limited coding capacity. The canine parvovirus (CPV), a member of the autonomous parvoviruses, has a genome of approximately 5 kb encoding two viral structural proteins, VP1 (84 kDa) and VP2 (67 kDa), and two non-structural proteins, NS1 (77 kDa) and NS2 (22 kDa) (Tsao et al., 1991; Wang et al., 1998). CPV NS1 is a replication protein with multiple functions including site-specific DNA binding, ATPase, helicase, and nickase activities (Niskanen et al., 2013, 2010). The double-stranded DNA binding of NS1 depends on the binding and hydrolysis of ATP (Niskanen et al., 2013, 2010). NS2 plays important role in viral replication (Naeger et al., 1990; Choi et al., 2005) and the chromosome region maintenance 1 protein (CRM1 or exportin 1) -mediated nuclear egress of minute virus of mice (MVM) capsids (Bodendorf et al., 1999; Eichwald et al., 2002; Miller and Pintel, 2002; Engelsma et al., 2008). Our recent studies revealed that CPV NS2 is associated with nuclear processes such as chromatin organization and DNA damage response (DDR), which most likely affect the progress of CPV replication (Mattola et al., 2022).

Parvovirus infection appears to modulate host cell cycle machinery to benefit viral replication. In early parvovirus infection, the activation of mitosis leads to the disintegration of the nuclear envelope (NE), which is likely followed by an enhanced nuclear entry of viral capsids (Porwal et al., 2013). Later in infection, DNA damage-induced G2/M cell cycle arrest and its regulation is important for the progress of infection. The cellular G2/M checkpoint, which controls entry into mitosis, is regulated by the cyclin-dependent kinase 1 (Cdk1) in association with cyclin B (Santamaría et al., 2007; Hochegger et al., 2008; Satyanarayana and Kaldis, 2009; Kasim Diril et al., 2012). The Cdk1-mediated activation of the G2/M checkpoint leads to mitotic disintegration of the NE and an increase in nuclear permeability (Feldherr and Akin, 1990; Dultz et al., 2009). DNA damage and specific inhibition of Cdk1 activity can prevent the G2/M phase transition which may induce apoptosis (Smits and Medema, 2001; Furusawa et al., 2012; Wu et al., 2013; Sunada et al., 2021). DNA damage-induced apoptosis is regulated by the nuclear import of cyclin B (Porter et al., 2003). In parvovirus infection, the proceeding of the viral life cycle results in cellular DNA damage, which activates DDR signaling pathways orchestrated by ATM-dependent kinases (Adeyemi et al., 2010; Luo et al., 2013; Adeyemi and Pintel, 2014). This elicits the G2/M cell cycle arrest, which ensures that MVM- and B19-infected cells do not proceed into mitosis, thereby maintaining nuclear integrity and allowing the prolonged assembly of new virions (Morita et al., 2003; Chen et al., 2010; Adeyemi and Pintel, 2014). Moreover, it has been shown that the replication centers, also known as autonomous parvovirus-associated replication (APAR) bodies of MVM, are associated with sites of cellular DNA damage (Majumder et al., 2018, 2017). This suggests that MVM exploits the proteins of cellular DDR machinery to facilitate its replication processes (Adeyemi et al., 2010; Adeyemi and Pintel, 2014; Cotmore and Tattersall, 2014). In addition to DDR response, parvoviruses use varied mechanisms to regulate the cell cycle (Chen and Qiu, 2010). For example, MVM induces a p21- and Cdk1-independent G2/M arrest (Adeyemi and Pintel, 2014). Infection of adeno-associated virus 2 (AAV2) causes a prolonged S phase associated with the ability of viral Rep78 protein to nick cellular DNA (Berthet et al., 2005). Infection with human parvovirus B19 results in G2 arrest by cytoplasmic retention of cyclins, preventing their entry into the nucleus and thus the progression into mitosis (Morita et al., 2001). Several studies have shown that, in addition to pre-mitotic cell cycle arrest, parvoviruses (e.g. CPV) induce DDR and promote apoptosis (Luo et al., 2013; Zhao et al., 2016). Both MVM and B19 induce caspase-dependent apoptosis in infected cells (Moffatt et al., 1998; Morita et al., 2003; Poole et al., 2004; Mincberg et al., 2011). Following rat parvovirus H-1 infection, there is an increase in intracellular reactive oxygen species leading to DNA double-strand breaks, cell cycle arrest, and caspase-mediated apoptosis (Ohshima et al., 1998; Rayet et al., 1998; Ran et al., 1999; Moehler et al., 2001; Hristov et al., 2010).

In DNA viruses, DNA damage pathways are activated in response to the expression of viral proteins, production of viral DNA, and virus-induced DNA damage (Davy and Doorbar, 2007; Weitzman et al., 2010; Luftig, 2014; Alekseev et al., 2020). The specific role of parvoviral NS proteins in the activation of DDR and cell cycle arrest at G2/M has remained partially undefined. However, the MVM NS1 protein induces cell cycle arrest (Anouja et al., 1997; op de Beeck et al., 2001), which has been suggested to be caused by the direct nicking of the host cell chromatin by the NS1. Unlike MVM, in parvovirus minute virus of canine (MVC) infection, the G2/M cell cycle arrest is independent of NS1 protein or viral replication but is rather induced by the single-stranded viral genome with terminal hairpin loops (Cotmore and Tattersall, 1994; op de Beeck and Caillet-Fauquet, 1997). NS1 of parvoviruses MVM, H-1, and B19 have cytotoxic effects on host cells and possess the ability to induce caspase-mediated apoptosis (Anouja et al., 1997; Chen et al., 2010; Daeffler et al., 2003; de Beeck et al., 1995; Hristov et al., 2010; Hsu et al., 2004; op de Beeck et al., 2001; op de Beeck and Caillet-Fauquet, 1997; Poole et al., 2004). In contrast, in the Aleutian mink disease virus (AMDV) the capsid proteins activate caspases and lead to apoptosis (Cheng et al., 2010).

To characterize the role of G2/M checkpoint regulation and apoptosis-induced changes in the nuclear egress of viral capsids, we used light microscopy approaches, including photobleaching, combined with advanced data analyses, and chromatin immunoprecipitation sequencing (ChIP–seq). Our observations support a general model that the egress of progeny capsid is facilitated not only by CRM1-mediated transport but also by G2/M checkpoint function and by apoptotic events affecting the NE permeability and nuclear egress of capsids.

## Materials and methods

### Cell lines and viruses

Norden laboratory feline kidney (NLFK) and human cervical carcinoma HeLa cells were cultured in Dulbecco`s modified Eagle medium (DMEM) supplemented with 10% fetal bovine serum (FBS), 1% non-essential amino acids, and 1% penicillin-streptomycin (Gibco, Thermo Fischer Scientific, Waltham, MA) at 37°C with 5% CO_2_. CPV type 2 was derived from an infectious plasmid clone p265 (Parrish, 1991) by transfection of NLFK as previously described (Parrish, 1991; Parker et al., 2001). The viruses were grown in NLFK cells, a virus-containing medium was collected, and the virus was concentrated by ultrafiltration.

### Plasmids

Experiments with importin α were conducted with importin-α-GFP transfected cells. Importin-α-GFP plasmid was a generous gift from Enrico Gratton (Irvine, CA, United States). Transfections were performed with TransIT 2020 transfection reagent (Mirus Bio LLC, Madison, WI, United States).

### Chemicals and antibodies

CRM1 inhibitor leptomycin B (LMB) was obtained from Abcam (Cambridge, United Kingdom) and it was used at a concentration of 100 ng/ml (Maroto et al., 2004). Cells were treated with LMB for 10 h. Cdk1 (RO-3306) and caspase 3 (Caspase-3 Inhibitor Z-DEVD-FMK**)** inhibitors were obtained from SelleckChem (Munich, Germany) and Bio-Techne (Minneapolis, MN), and they were used at a final concentration of 10 µM and 50 μM, respectively. Cells were treated with the Cdk1 inhibitor for 7 h and with the caspase 3 inhibitor for 16 h. Rabbit Ab against CPV VP2 capsid protein and mouse monoclonal antibody (MAb) which recognizes the intact capsids (Weichert et al., 1998) was used at a concentration 4–10 μg/ml. The anti-NS1 MAb was a generous gift from Dr. Caroline Astell (University of British Columbia, Vancouver, Canada) (Yeung et al., 1991) (6.7 μg/ml). Full CPV capsids have their 5′-end of the DNA (Wang and Parrish, 1999) and N-terminal end of VP2 (Paradiso et al., 1982; Parrish et al., 1991)exposed outside the capsid. Here full capsids were detected with a rabbit antibody (rAb) against a peptide from the VP2 N-terminal domain (residues 2–19: CDGAVQPDGGQPAVRNER) (Casal et al., 1995) (2.7 μg/ml). Commercial antibodies obtained from Abcam (Cambridge, United Kingdom) against indicated proteins were: CRM1 (rabbit polyclonal Antibody, rAb, ab24189), γ-H2AX (recombinant Alexa Fluor^®^ 647 H2A.X anti-gamma phospho S139 rabbit monoclonal antibody, rMAb, ab195189 or anti-gamma H2A.X (phospho S139) antibody, rAb, ab2893), cyclin B1 (recombinant Alexa Fluor^®^ 555 Cyclin B1 rMAb, ab214381 or anti-Cyclin B1, ab2949), Cdk1 (recombinant Anti-Cdk1 rMAb, 133327)importin β (mMAb, KPNB1, ab2811), Ran (rAb, ab53774), and lamin B1 (anti- (rAb, ab16048). The primary antibodies were followed by goat anti-mouse or anti-rabbit Alexa448, Alexa546, Alexa 633, or Alexa 647 conjugated secondary Abs (Thermo Fisher Scientific, Waltham, MA). Commercial antibodies were used according to concentrations recommended by the manufacturer.

### Confocal microscopy and image analyses

For immunofluorescence, NLFK and HeLa cells were cultured on glass coverslips and infected with CPV. Cells were fixed at 24 h post infection (hpi), and 30 hpi in 4% paraformaldehyde for 12 min and permeabilized with 0.1% Triton X-100 in phosphate-buffered saline supplemented with 1% bovine serum albumin. Immunolabeling with primary antibodies was followed by anti-mouse or anti-rabbit secondary antibodies. Nuclei were stained with Pro-Long Diamond anti-fade media with DAPI or Prolong Glass antifade mountant with NucBlue (Thermo Fisher Scientific). Imaging of immunolabeled samples was performed with Olympus FluoView FV1000 (Olympus, Tokyo, Japan), Nikon A1R (Nikon, Tokyo, Japan), and Leica TCS SP8 FALCON (Leica microsystems, Mannheim, Germany) laser scanning confocal microscopes. For Olympus microscopic parameters were the following: DAPI was excited with a 405 nm diode laser and a 450/50 nm band-pass filter was used to detect the fluorescence. Alexa 488 and Alexa 633 were excited with 488 nm argon and 633 nm He-Ne lasers, and the fluorescence was collected with a 500–555 nm slit-based emission filter and 647 nm long-pass filter, respectively. For Nikon, the following microscopic parameters were used. DAPI was excited with a 405 nm diode laser collected with a 513/30 nm band-pass filter. Alexa 488 was excited with a 488-nm argon laser, and fluorescence was collected with a 515/30 nm band-pass filter. Alexa 546 was exited with a 561 nm sapphire laser and detected with a 595/50 band-pass filter, and Alexa 633 was excited with a 642 nm diode laser and detected with a 660 nm long-pass filter. Microscopy images with Leica TCS SP8 FALCON were acquired as follows: DAPI and Nucblue were excited with a 405-nm diode laser and the fluorescence was monitored between 415–495 nm. Alexa 488, Alexa 546 or Alexa 555, and Alexa 647 were excited with 499 nm, 557 nm, and 653 nm wavelengths of pulsed white light laser (80 MHz). The emission detection range was 505–561 nm for Alexa 488 and 568–648 nm for Alexa 546/555 and 663–776 for Alexa 647. The image size varied from 512 × 512 to 1700 × 1700 pixels with a pixel size between 50–100 nm in the x- and y-directions. For the microscopy image stack, the step size of the images was between 120–333 nm in the *z*-direction. In the large field microscopy images used for the classification of infected cells, the image size was either 1024 × 1024 with a pixel size of approximately 210 nm or 4700 × 4700 with a pixel size of approximately 415 nm. For the images used for quantitative image analyses, the laser powers were fixed.

To analyze the nuclear and cytoplasmic intensities as well as the distributions of fluorescent labels concerning the nuclear border, the nuclei were segmented using Otsu’s method (Otsu, 1979) for the DNA stain channel. The part of the image that was not within the segmented nuclei was assumed to be within the cytoplasm. This causes the cell exterior to be included in the cytoplasm calculations, but it does not affect the calculated total intensities since the label signals were very weak outside cells. To calculate the intensity of labels concerning the nuclear border, the Euclidean distance to the nuclear border was determined for each pixel and the mean pixel intensity was calculated for ranges of increasing distances. In the analyses of cells with nuclear or cytoplasmic cyclin localization, the cells were grouped so that if the total cyclin signal was higher in the nucleus than in the cytoplasm the cell was classified as belonging to the nuclear cyclin group. Otherwise, it was classified as having cytoplasmic cyclin. In every analysis, the statistics were calculated over all the imaged cells. Student’s t-test, Games-Howell test, and chi-square test were used to evaluate the statistical significance.

### FLIP

NLFK cells stably expressing enhanced green fluorescent protein (EGFP) were used for fluorescence loss in photobleaching (FLIP) experiments. Non-infected and infected cells were incubated in the presence or absence of Cdk1 and caspase 3 inhibitors for 7 and 16 h, respectively, before the FLIP analysis at 24 hpi. The cells were imaged at 37°C and 5% CO2 with Nikon A1R laser scanning confocal microscope. Before the experiments, cells were treated for 15 min with 1 μg/ml Hoechst 33342 (Thermo Fisher Scientific) to visualize the nuclei of the cells and identify infected cells by prominent marginalization of host chromatin characteristic of CPV infection. A circular area of 3 µm in diameter in the cytoplasm was repeatedly photobleached with a 488 nm laser line using full laser power. Images of the whole cell and a neighboring reference cell were taken before and between the photobleaching pulses for 175 s. To analyze NE permeability to EGFP, fluorescence intensities from the nucleus of the photobleached cell and the reference cell were extracted. The values were normalized to pre-bleach intensities and corrected for the loss of fluorescence resulting from the imaging procedure with fluorescence intensities from the reference cell. Student’s t-test was used to analyze the statistical significance of the differences in the averaged nuclear fluorescence half-lives between samples.

### ChIP-seq and ChIP-qPCR

ChIP-seq and ChIP-qPCR were performed according to Mäntylä et al. (Mäntylä et al., 2016). ChIP-seq sequence reads were re-aligned with the updated cat reference genome (Felis_catus 9). Analysis was limited to enrichment in regions surrounding transcription start sites (TSS, TSS between -2000 and 1,500 bp). GO biological process term enrichment analyses were performed with http://geneontology.org/. The ChIP-seq data reported in this paper have been previously deposited in the NCBI Gene Expression Omnibus (GEO) database under accession number GSE77785.

## Results

### Full capsids exit the nucleus when nuclear export factor CRM1 is inhibited

As we have described earlier, the CRM1-mediated nuclear export does not fully explain the nuclear egress of all CPV progeny capsids (Mattola et al., 2022). Here, we analyzed the nuclear exit of full (DNA-containing) capsids in the presence of CRM1 inhibitor LMB. The full capsids with exposed N-terminal end of VP2 (Paradiso et al., 1982; Parrish et al., 1991) were detected with an antibody against the VP2 N-terminal domain.

**FIGURE 1:**
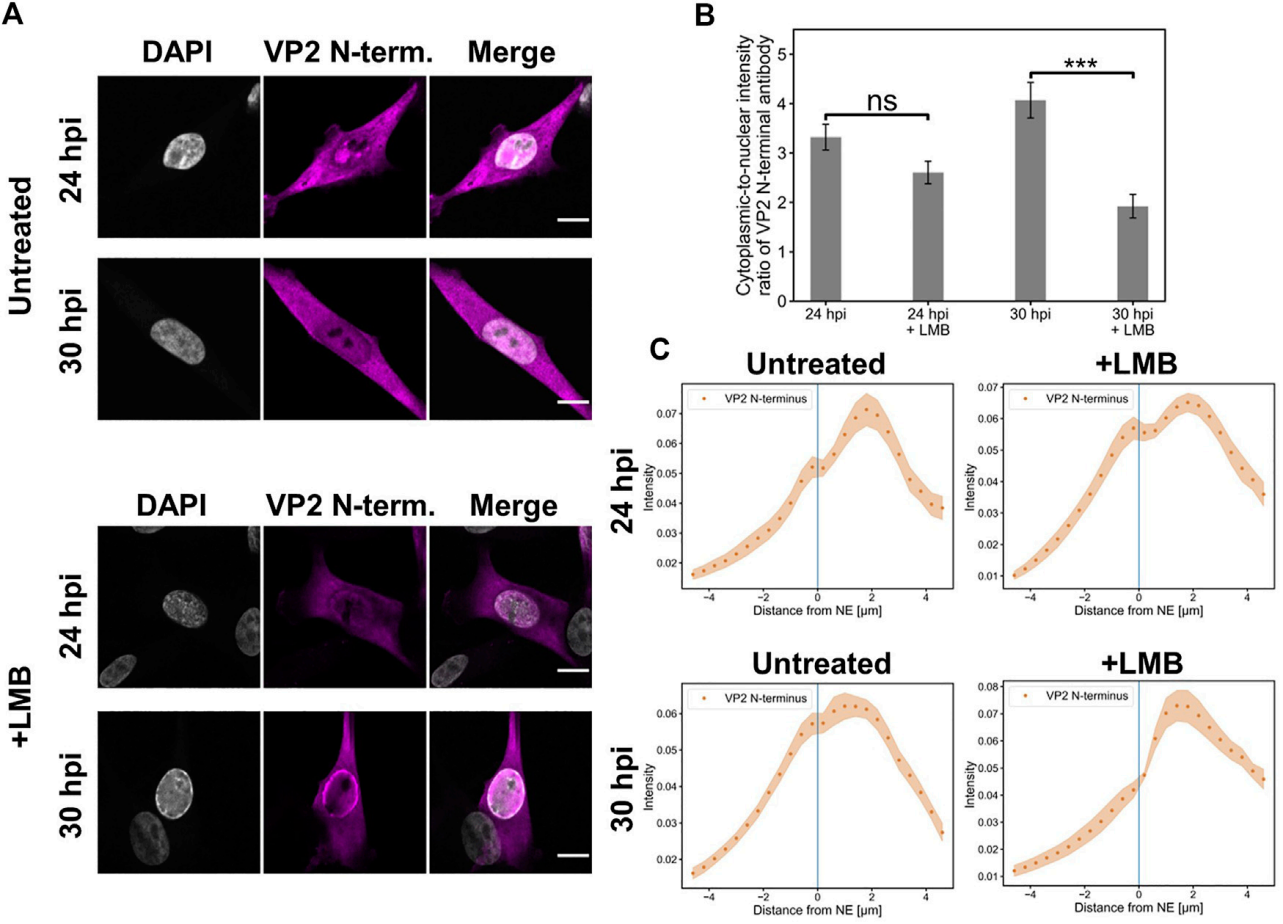
Full capsids egress the nucleus in the presence of leptomycin B **(A)** Representative confocal images of intracellular distribution of full (DNA-containing) capsids (magenta) and DAPI (blue) in NLFK cells in the presence and absence of LMB at 24 and 30 hpi. Capsids were detected with an antibody recognizing the N-terminal end of VP2 exposed outside the full capsids. LMB (100 ng/ml) was added at 10 hpi Scale bars, 10 µm. **(B)** Quantitative analysis showing the cytoplasmic-to-nuclear ratio of full capsids in non-treated and LMB-treated cells. The ratios were calculated from summed intensities of full capsid antibody in the cytoplasm and nucleus. Statistical significances were determined using Student´s *t*-test. The significance values shown are denoted as *** (*p* < 0.01) and non-significant as ns (*p* > 0.05). **(C)** Nuclear and cytoplasmic distribution of full capsids as a function of increasing distance from the NE in the absence and presence of LMB at 24 and 30 hpi. The shaded areas around the data points represent the standard error of the mean (*n* = 16).

Our confocal microscopy imaging demonstrated that the nuclear egress of full CPV capsids into the cytoplasm was slightly decreased at 24 hpi in the presence of LMB in comparison to the untreated infected NLFK cells (Figure 1A). Quantitative analysis of the cytoplasmic-to-nucleus ratio showed a moderate but non-significant LMB-induced reduction of cytoplasmic capsids at 24 hpi in comparison to non-treated cells, and a significant decrease at 30 hpi (Figure 1B). However, at both time points, nuclear escape of capsids was detected also in the presence of LMB. The analysis of the cellular distribution of viral capsids in untreated and LMB-treated infected cells at 24 and 30 hpi indicated that the number of cytoplasmic capsids was the highest close to the NE and decreased towards the plasma membrane. In the nucleus, the majority of capsids accumulated close to the NE in the absence and presence of LMB both at 24 and 30 hpi. However, in the LMB-treated cells, specifically at 30 hpi, the capsids were more closely associated with the NE than in non-treated cells. Full capsids were not detected in infected cells earlier at 14 hpi.

Altogether, the LMB treatment cannot fully inhibit the nuclear egress of capsids, thereby suggesting that CRM1-independent pathways are involved in the nuclear egress of full capsids.

### NE permeability is significantly increased in infection

The nuclear egress of capsids could be explained by a partial loss of the nuclear permeability barrier. In non-infected cells, NE permeability is increased at the early stages of mitosis (Terasaki et al., 2001; Beaudouin et al., 2002; Burke and Ellenberg, 2002) in apoptosis (Coleman and Olson, 2002; Kopeina et al., 2018; Lindenboim et al., 2020), and during nuclear envelope ruptures (Denais et al., 2016). In mitosis, NE breakdown is regulated by kinases such as Cdk1 (Santamaría et al., 2007). During the apoptotic processes, caspases promote an increase in NE permeability by cleaving nuclear pore complexes and the nuclear lamina Kopeina et al., 2018; Lindenboim et al., 2020). Moreover, Cdk1 is also linked to the regulation of apoptosis (Castedo et al., 2002) and caspase 3 has a potential role in mitosis (Swe and Sit, 2000; Hsu et al., 2006; Hashimoto et al., 2011; Lee et al., 2011).

**FIGURE 2:**
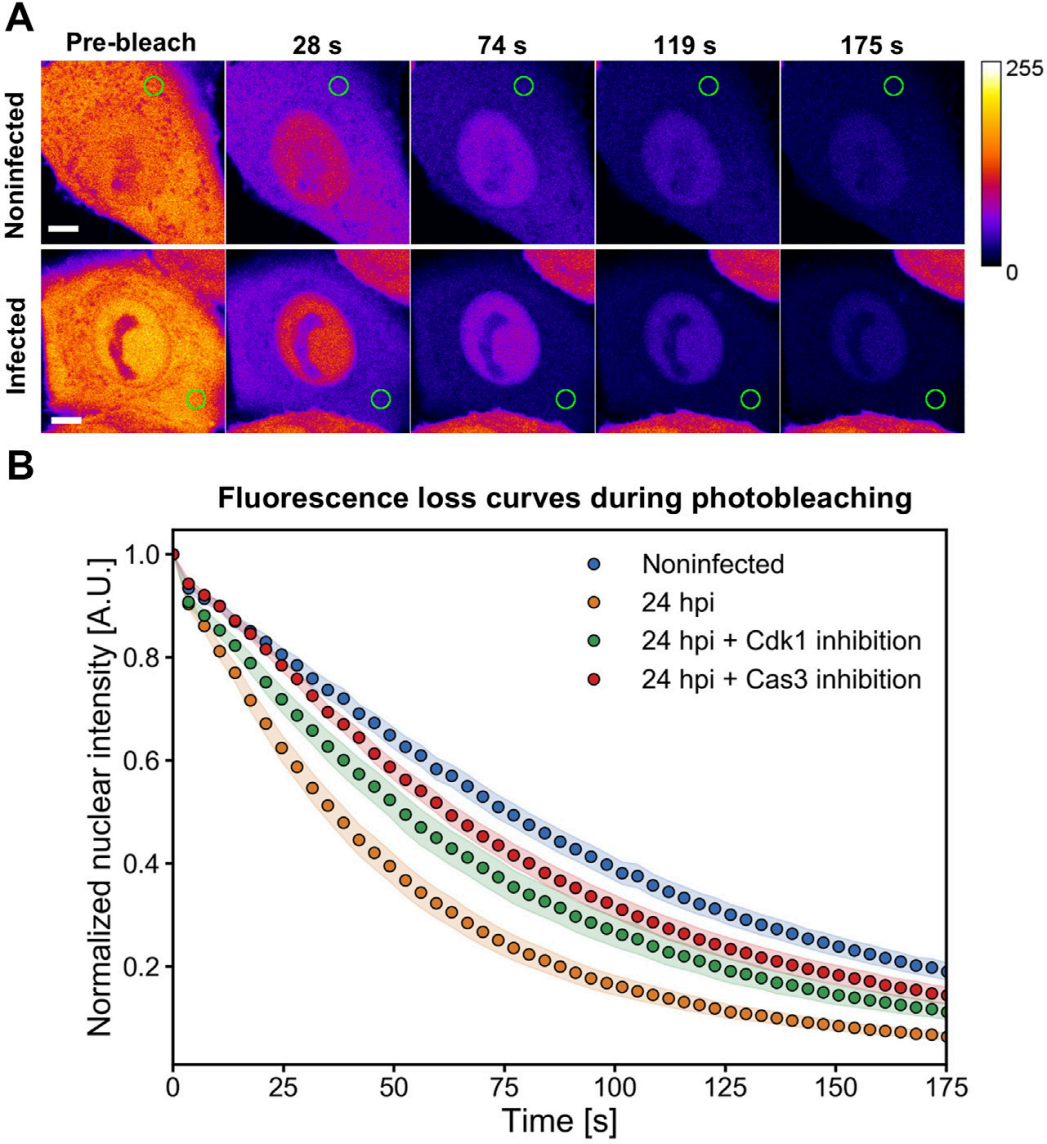
Nuclear envelope permeability is increased in infection and restored upon exposure to Cdk1 and caspase inhibitors. FLIP analyses of nuclear envelope permeability in infected cells at 24 hpi. **(A)** Infected and non-infected NLFK cells stably expressing EGFP were photobleached in the cytoplasm as indicated by the green circle. The fluorescence images before photobleaching (time = 0 s) and four different time points during photobleaching are shown. A calibration bar for pseudo coloring is shown. Scale bars, 5 µm. **(B)** Nuclear envelope permeability of infected cells at 24 hpi in the presence and absence of inhibitors of Cdk1 (RO-3306) and apoptosis (caspase 3 inhibitor). Quantification of fluorescence loss at the nucleoplasmic region of interest during a 175 s period of continual photobleaching of the cytoplasm. The fluorescence loss curves showing the relative fluorescence intensity are presented for non-infected untreated control cells (blue) (*n* = 12), infected cells (orange) (*n* = 17), and infected cells in the presence of Cdk1 (red) (*n* = 13), and caspase 3 (green) (*n* = 14) inhibitors. The shaded areas around the data points represent the standard error of the mean.

To study further the possible association between capsid egress and NE leakage, we investigated NE permeability at a late stage of infection (24 hpi). We used FLIP to determine the transport rate of freely diffusible EGFP through the NE (Hoffmann-rohrer et al., 2003; Lippincott-Schwartz et al., 2003). The results show that the repeated cytoplasmic EGFP bleaching led to a faster depletion of nuclear fluorescence of infected cells at 24 hpi in comparison to non-infected cells (Figures 2A,B). The fluorescence half-life in the nucleus was significantly (*p* < 0.001) decreased from 77 ± 4 s to 39 ± 3 s (mean ± the standard error of the mean). This implies that CPV infection is accompanied by an increase in NE permeability. We next studied the potential role played by the key effector proteins of mitosis and apoptosis, Cdk1 and caspase 3, using FLIP analysis for NE leakiness in the presence of Cdk1 and caspase 3 inhibitors. The analysis showed that the infection-induced increase in NE permeability was partially reversed upon exposure to the inhibitors (Figure 2B), leading to significantly increased fluorescence half-life in the nucleus from 39 ± 3 s to 55 ± 5 s (*p* < 0.01) and 66 ± 6 s (*p* < 0.001) for Cdk1 inhibitor and caspase 3 inhibitor, respectively. Notably, inhibition of Cdk1 or caspase 3 failed to fully restore NE permeability to the level of non-infected cells. This suggests that neither mitosis nor apoptosis alone is responsible for NE leakage. In non-infected cells treated with inhibitors, NE permeability remained similar to the untreated cells (Supplementary Figure S1).

Together, our results suggest that Cdk1- and caspase 3 -mediated mitotic and apoptotic pathways may affect NE permeability in infection, and thereby most likely influence the nuclear egress of progeny capsids.

### Nuclear egress of capsids is more effective in cells with perinuclear accumulation of DNA damage

As parvovirus infection progresses, it induces cellular DNA damage and evokes the DDR (Adeyemi et al., 2010; Luo et al., 2011a; Luo et al., 2011b). The localization of DDR can be detected by staining the phosphorylated histone variant, γ-H2AX, formed from H2AX in a response to double-strand break formation (Kinner et al., 2008). DNA damage induces chromatin decondensation, which leads to an enhancement of nuclear molecular diffusion (dos Santos et al., 2021). Here, we examined the effect of DNA damage-induced chromatin changes on the nuclear egress of CPV capsids.

Earlier MVM and CPV studies have demonstrated that at late times of infection the pattern of capsid localization progresses through characteristic stages. These stages include localization of progeny capsids in the nuclear replication center, accumulation of capsids to the nuclear periphery sometimes accompanied by the presence of cytoplasmic capsids, and egress phase with major cytoplasmic localization of egressed capsids. The intracellular distribution of capsids varies between individual cells, thereby reflecting cell-specific variation in the progression of infection (Ruiz et al., 2011; Mihaylov et al., 2014; Mäntylä et al., 2016). To analyze the nuclear distribution of γ-H2AX at 24 hpi, we divided infected cells into two classes: a replication center class showing capsid accumulation into the central nuclear region and an egress class showing capsid localization both in the nuclear periphery and in the cytoplasm.

**FIGURE 3:**
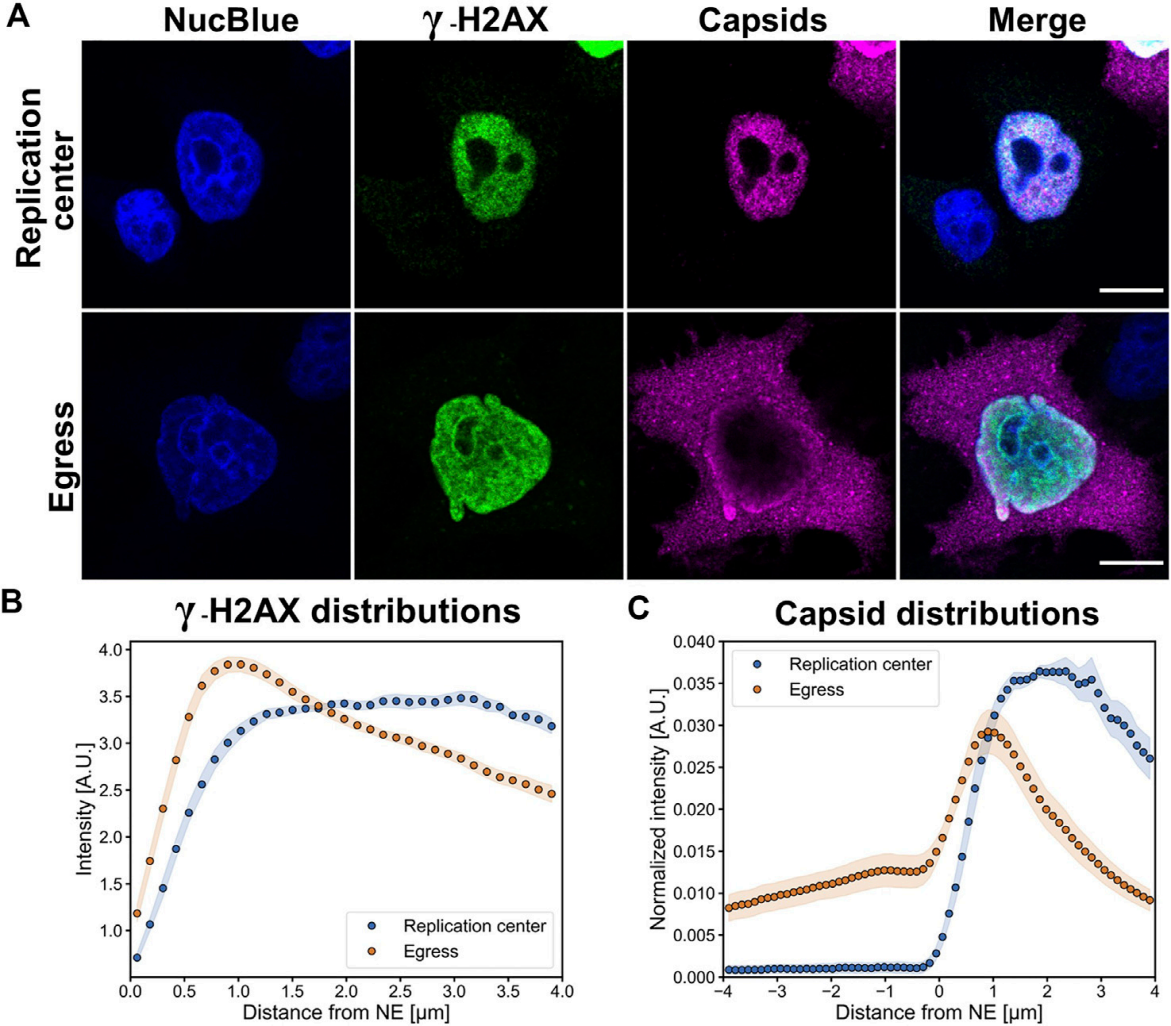
Perinuclear localization of DNA damage is accompanied by the nuclear escape of viral capsids. **(A)** Representative confocal images showing the distribution of DNA damage (γ-H2AX, green) and viral capsids (magenta) in Hela cells at 24 hpi. The infected cells were divided into two main infection stages, defined by nuclear accumulation of capsids (replication center) and localization both to the nucleus and cytoplasm (egress). The cells were immunolabeled with antibodies against intact capsids and γ-H2AX, and blue corresponds to NucBlue staining. Scale bars, 10 µm. **(B)** Quantitative analyses of the distribution of nuclear γ-H2AX and **(C)** cellular localization of capsids in replication center and egress class cells as a function of the distance from the nuclear envelope. The negative *x*-axis corresponds to the cytoplasmic and the positive to the nuclear side of the nuclear envelope. The shaded areas around the data points represent the standard error of the mean (*n* = 10).

Confocal images showed that in the replication center class γ-H2AX was mostly distributed homogenously throughout the nucleus with a nucleolar exclusion, whereas in the egress class γ-H2AX was concentrated close to the NE and around the nucleoli (Figure 3A). Host cell chromatin was distributed both near the NE and nucleoli (Figs 3A and S2). Distance analyses showed that γ-H2AX was distributed throughout the nucleoplasm both in cells with nuclear accumulation of capsids and in cells with egressed capsids, however, in the egress class cells there was a clear increase in γ-H2AX intensity toward the NE (Figure 3B). It is important to note that an increase in the cytosolic amount of capsid label (Figure 3C) correlated with the perinuclear localization of damage induces chromatin decondensation γ-H2AX. As shown for herpesvirus, transport through the host chromatin can be a rate-limiting step in the nuclear egress of capsids (Aho et al., 2021). Our results imply that cellular DNA damage and DDR-related events could enhance the nuclear exit of CPV capsids.

### The infection leads to nuclear accumulation of cyclin B1 in late-infection

In non-infected cells, the G2/M activation is accompanied by cytoplasmic interaction of cyclin B1 and Cdk1, which leads to nuclear translocation of the cyclin B1-Cdk1 complex (Hagting et al., 1999; Yang et al., 2001; Porter and Donoghue, 2003; Miyazaki and Arai, 2007). Nuclear accumulation of cyclin B1 is also detected in DNA damage-induced apoptosis at the G2/M checkpoint (Porter et al., 2003). In parvovirus-infected cells, virus-induced DDR results in cytoplasmic retention of cyclins during G2 arrest (Morita et al., 2001). Notably, in CPV infection the number of cells in the G2/M phase is relatively high at the late stages of infection (Nykky et al., 2010). To study the possible involvement of the G2/M checkpoint activation in infected cells, we analyzed the intracellular distribution of cyclin B1. Specifically, we were interested in observing the nuclear localization of cyclin B1 in cells with infection-induced DDR.

**FIGURE 4:**
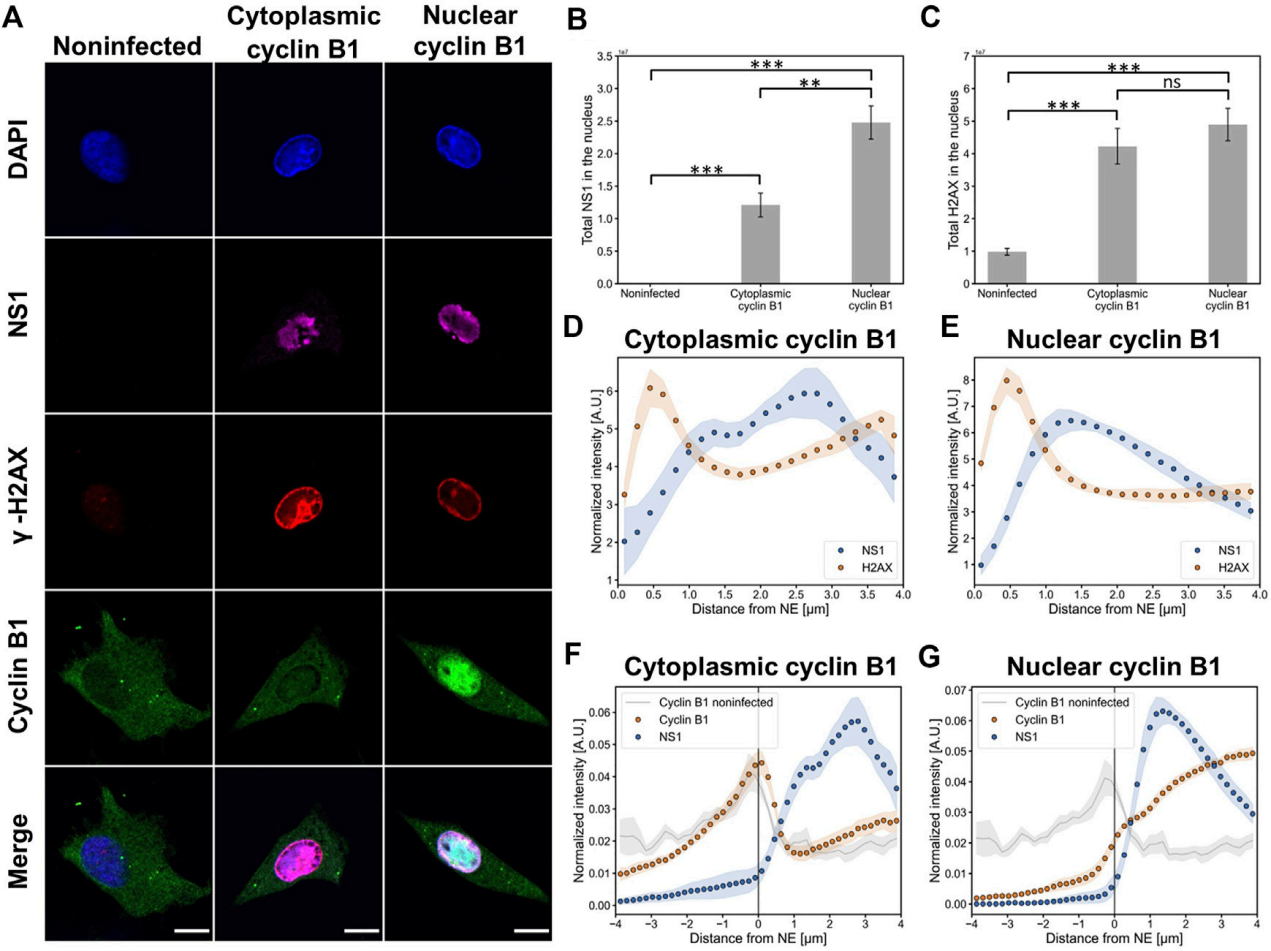
Infection induces DNA damage, and the increase of nuclear NS1 is accompanied by nuclear localization of cyclin B1 **(A)** Representative confocal images of nuclear and cytoplasmic localization of cyclin B1 in non-infected and infected cells at 24 hpi. NLFK cells were immunolabeled with antibodies against the viral NS1 protein (magenta), DNA damage marker γ-H2AX (red), and cyclin B1 (green). Blue corresponds to DAPI staining. Scale bars, 10 µm. Quantitative analyses of the amount of **(B)** NS1 and **(C)** γ-H2AX in the nuclei of non-infected (*n* = 10) and infected cells with cytoplasmic (*n* = 17) and nuclear (n = 9) cyclin B1. Statistical significances were determined using Games-Howell test. StatisticalSignificant differences are denoted as *** (*p* < 0.001) and non-significant as ns. The error bars show the standard error of the mean (SEM). Analyses of the relative amount of nuclear NS1 (blue) and γ-H2AX (orange) as a function of the distance from the nuclear envelope in infected cells with **(D)** cytoplasmic and **(E)** nuclear cyclin B1. Intracellular distribution of NS1 (blue) and cyclin B1 (orange) in infected cells with **(F)** cytoplasmic and **(G)** nuclear cyclin B1. Cyclin distribution (grey) in non-infected cells is also shown. The 2D nuclear boundary reconstruction located at x = 0 was based on the distribution of DAPI-labeled chromatin. The shaded areas around the data points represent SEM.

Our confocal images showed that at 24 hpi the main localization of cyclin B was either in the nucleus or in the cytoplasm. Approximately 30% (*n* = 9) of CPV-infected NLFK cells (*n* = 26) showed nuclear localization of cyclin B1. Viral infection was confirmed by the viral NS1 protein staining and by marginalized host cell chromatin, and DDR activation was detected by γH2A.X staining (Figure 4A). Quantitative analysis showed that in the presence of nuclear cyclin B1 the amount of viral NS1 was significantly increased in comparison to cells with cytoplasmic cyclin B1 (Figure 4B), whereas the amounts of γH2A.X in the presence of nuclear or cytoplasmic cyclin B1 were relatively similar (Figure 4C). Comparison between distributions of chromatin and γH2A.X as a function of the distance from the NE demonstrated that both in cells with cytoplasmic or nuclear cyclin B1, both chromatin (Fig S3A) and γH2A.X were accumulated close to the NE (Figures 4D,E), however, the amount of γH2A.X was increased in the presence of nuclear cyclin B1 (Fig S3B). Further distance analyses demonstrated that in cells with cytoplasmic cyclin B1 NS1 was localized in the nucleoplasmic area surrounded by the perinuclear γH2A.X (Figure 4D). In contrast, in cells with nuclear cyclin B1, NS1 was concentrated towards the γH2A.X layer (Figure 4E). Finally, the distribution analyses of cyclin B1 and NS1 demonstrated that in cells with mainly a cytoplasmic localization of cyclin B1, there was a nuclear fraction of cyclin B1 accumulated close to the NE, whereas NS1 was mostly localized into the center of the nucleus (Figure 4F). In cells with nuclear accumulation of cyclin B1, it was distributed throughout the nucleoplasm, however, with a slight increase toward the center of the nucleus. At the same time, NS1 remained in the nucleoplasm close to the perinuclear γH2A.X (Figure 4G).

Together, our experiments demonstrated that at 24 hpi infected cells showed differential localization of cyclin B1, both in the nucleus and in the cytoplasm. One-third of the infected cells had a nuclear accumulation of cyclin B1. These cells showed an increase in the amount of NS1, which suggests that these two phenomena might be connected. Nuclear localization of cyclin B1 implies that the infection-imposed G2/M block, represented by a cytoplasmic localization of cyclin B1, is not fully maintained at the late stages of infection. Alternatively, nuclear accumulation of cyclin B1 could be an indicator of cyclin B1–dependent apoptosis (Porter et al., 2003).

### An increase in nuclear cyclin B1 is accompanied by enhanced capsid egress

In non-infected interphase cells, cyclin B1 shuttles between the nucleus and the cytoplasm, and it is accumulated into the nucleus only after activation of the G2/M checkpoint (Lindqvist et al., 2007; Gavet and Pines, 2010; Müllers et al., 2014). Nuclear accumulation of cyclin B1 is also a key regulator in the cellular decision to undergo apoptosis in response to DNA damage (Porter and Donoghue, 2003).

**FIGURE 5:**
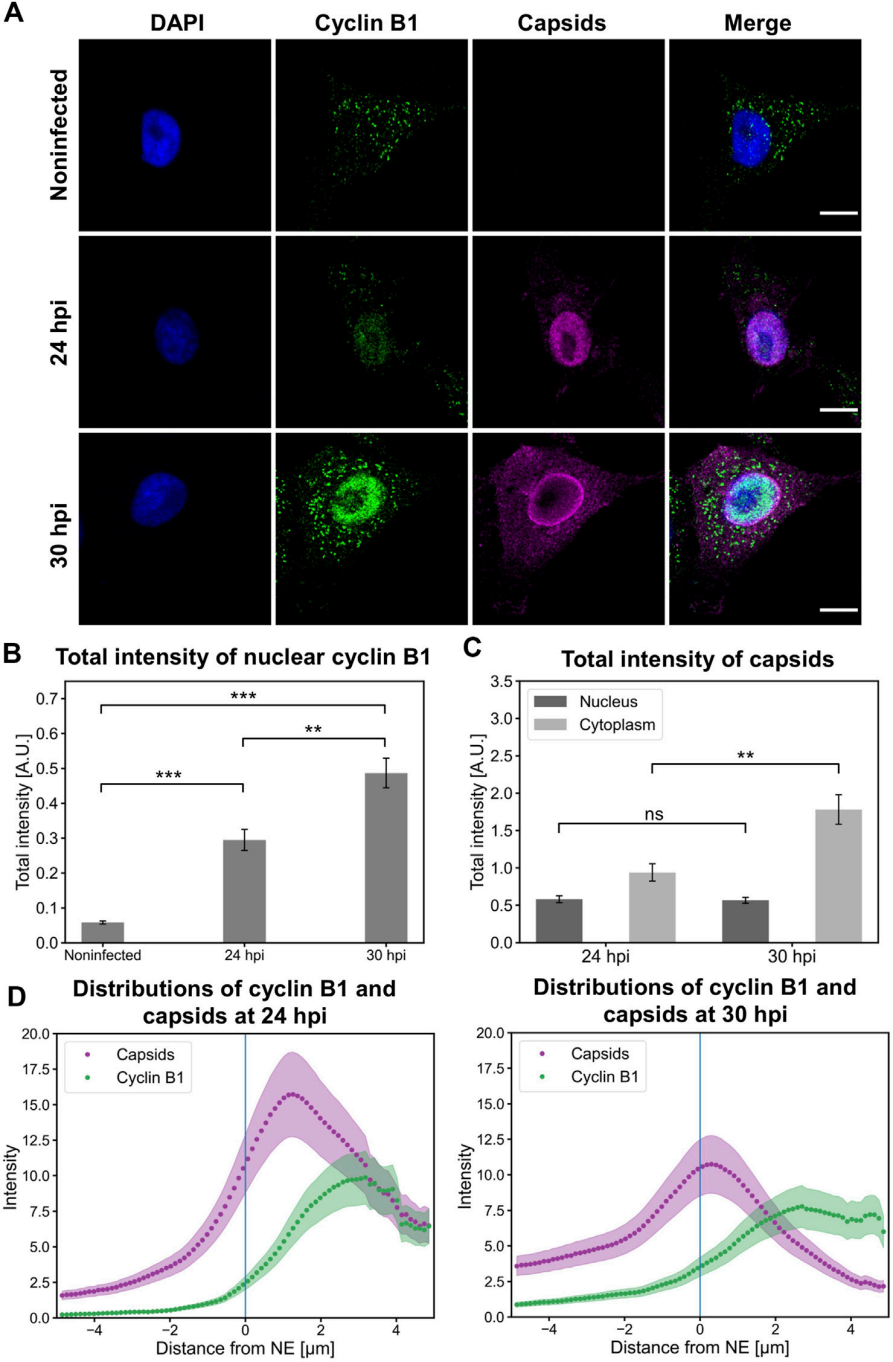
Nuclear accumulation of cyclin B1 is accompanied by the nuclear release of capsids. **(A)** Representative images showing the intracellular distribution of viral capsids and cyclin B1 in non-infected and infected NLFK cells at 24 and 30 hpi. The cells were immunolabeled with antibodies against cyclin B1 (green) and intact capsids (magenta). Blue corresponds to DAPI staining. Scale bars, 10 µm. **(B)** Total fluorescence intensities of nuclear cyclin B1 in non-infected and infected cells at 24 and 30 hpi and **(C)** total fluorescence intensities of capsids in the nucleus and the cytoplasm. The error bars show the standard error of the mean. Statistical significances were determined using Games-Howell test. The significance values shown are denoted as *** (*p* < 0.001) or ns (not significant) (*n* = 28). **(D)** Distributions of cyclin B1 and capsids as a function of distance from the NE (blue line) in infected cells at 24 and 30 hpi (n = 28). The shaded areas around the data points represent the standard error of the mean.

Here, we investigated the intracellular localization of capsids in NLFK cells together with cyclin B1. Nuclear retention of cyclin B1 (Figure 5A) in infected cells was further confirmed by total fluorescence intensity analysis. Infected cells showed a significant increase in the amount of nuclear cyclin B1 at 24 hpi compared to non-infected cells. Moreover, a significant increase in the amount of nuclear cyclin B1 was detected at 30 hpi (Figure 5B). This was consistent with the experiments described in Figure 4 and supports the model that upon infection a part of the infected cells enter mitosis. Nuclear amounts of nuclear capsid label did not change between 24 and 30 hpi, which suggests that egressing capsids were balanced by increased production of progeny viral capsids as verified in Figure 5C. Increasing nuclear cyclin B1 retention at 30 hpi was accompanied by capsid accumulation at the nuclear periphery (Figure 5A) and significant acceleration of capsid egress into the cytoplasm in comparison to 24 hpi (Figure 5C). Distribution analysis of capsids and cyclin B1 revealed that at 24 hpi capsids were more clearly retained in the nucleus whereas at 30 hpi the capsid distribution was more even throughout the nucleus and the cytoplasm. Cyclin B1 was mainly localized into the nucleus in infected cells with a small variation in distribution (Figure 5D).

Taken together, capsids escape from the nucleus in late infection when cyclin B1 is accumulated in the nucleus. The nuclear accumulation of cyclin B1 supports our overall model suggesting that G2/M checkpoint activation and associated apoptotic events enhance viral capsid egress out of the nucleus.

### Cdk1 is imported into the nucleus in infection and inhibition of Cdk1 activity leads to a decrease in nuclear egress of capsids

**FIGURE 6:**
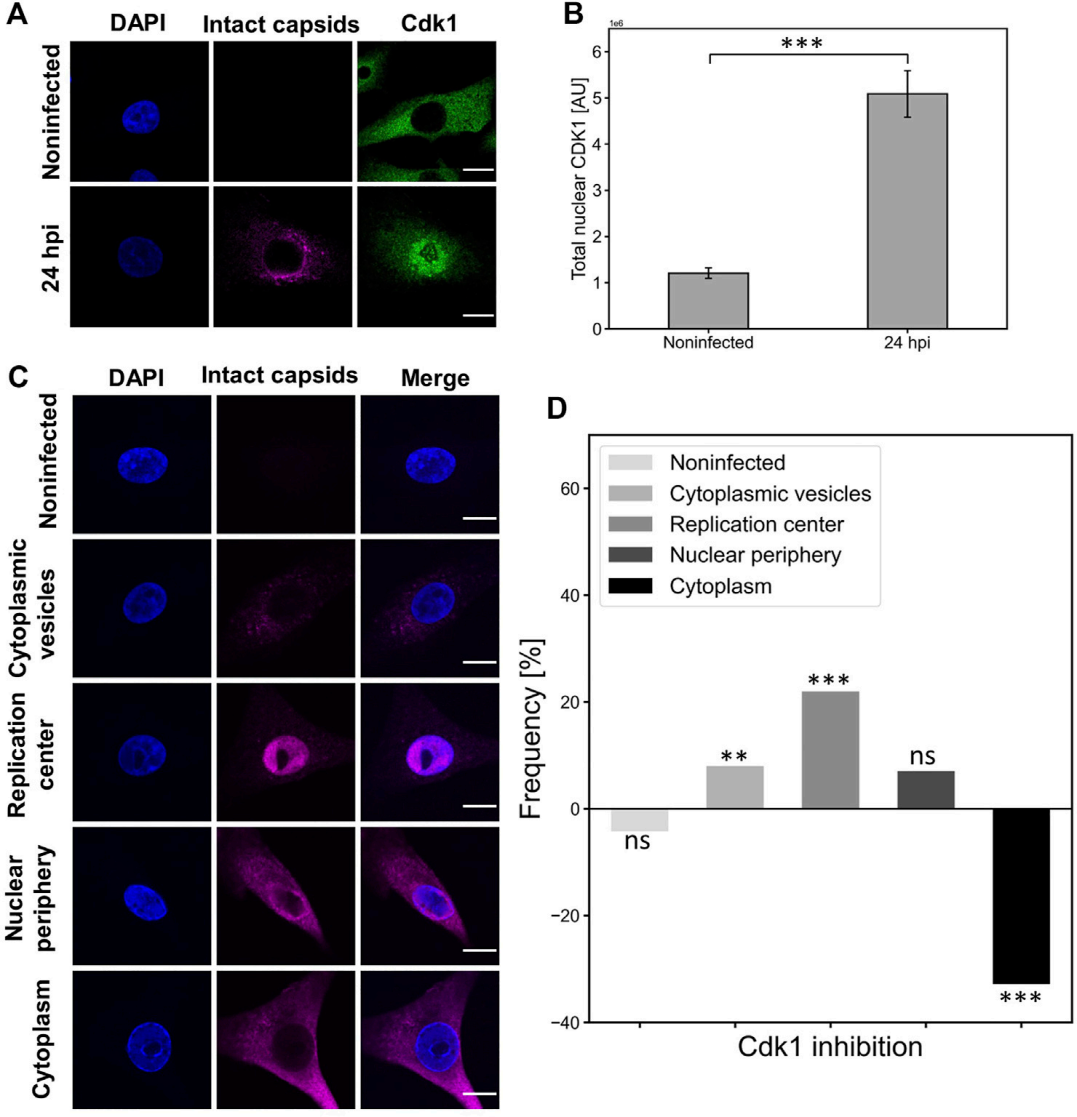
Cdk1 is accumulated into the nucleus in infection and its inactivation decreases the nuclear egress of capsids. **(A)** Representative confocal images show the distribution of capsids (magenta) and Cdk1 (green) in NLFK cells at 24 hpi. The cells were immunolabeled with antibodies against intact capsids and Cdk1, and blue corresponds to DAPI staining. Scale bars, 10 µm. **(B)** Total nuclear fluorescence intensities of antibody-labeled Cdk1 in non-infected and infected cells (*n* = 28). Statistical significances were determined using Student´s *t*-test. Significant differences are denoted as *** (*p* < 0.001). The error bars show the standard error of the mean (SEM). **(C)** The intracellular variation in the cellular distribution of capsids at 24 hpi. Five capsid localization classes are shown: non-infected, cytoplasmic vesicles, replication center, nuclear periphery and cytoplasm (nuclear periphery), and cytoplasm. The cells were immunolabeled with antibodies against intact capsids (magenta) and blue corresponds to DAPI staining. Scale bars, 10 µm. **(D)** The effect of Cdk1 inhibitor RO3306 treatment on the incidence of capsid distribution classes. The horizontal line at value 0 represents capsid distribution in untreated infected cells at 24 hpi, and positive and negative values represent treatment-induced increase and decrease in the frequency of the capsid distribution classes. Significant differences tested with the chi-square test are denoted as *** (*p* < 0.001), ** (*p* < 0.01), and the non-significant as ns (*p* > 0.05).

Nuclear accumulation of cyclin B1 during the G2/M checkpoint activation is accompanied by nuclear localization of Cdk1(Gavet and Pines, 2010; Morgan, 2007; Schmitt et al., 2007). To further investigate the association between Cdk1 distribution and nuclear egress of capsids, we analyzed infected NLFK cells at 24 hpi. We observed that the amount of nuclear Cdk1 was increased in infected cells at the same time as the nuclear egress of viral capsids (Figure 6A). Quantitative analyses of Cdk1 distribution confirmed the infection-induced significant nuclear accumulation of Cdk1 at late infection (Figure 6B). We next sought to analyze how the inhibition of Cdk1 affects the nuclear egress of capsids. In these analyses, we used a classification based on capsid localization into cytoplasmic vesicles (capsids in endosomal vesicles), replication center (capsids located in the nucleus), nuclear periphery (capsids in the nucleoplasm and cytoplasm), and cytoplasm (empty nucleus after capsid egress) (Figure 6C). The intracellular distribution of capsids was monitored in the presence of a selective Cdk1 inhibitor RO3306, which reversibly arrests human cells at the G_2_/M border (Vassilev, 2006). Our findings showed that the inhibition of Cdk1 led to a clear increase in the amount of capsid label retained in the nucleus, and to a slight increase in capsid label found near the NE. Importantly, a significant decrease in the amount of capsid label translocated into the cytoplasm was detected (Figure 6D).

Altogether, this indicated that Cdk1 is located in the nucleus in infected cells and Cdk1 activity has a role in the nuclear egress of CPV capsids. The strong correlation between Cdk1 inhibition and a decrease in the cytoplasmic amounts of capsid label is consistent with our results above showing that G2/M checkpoint-mediated NE leakage is required for the nuclear egress of capsids in late infection.

### The infection leads to apoptotic redistribution of nuclear transport factors

Early apoptosis leads to nucleoporin cleavage, an increase in passive diffusion through nuclear pore complexes, and nucleocytoplasmic relocalization of active nuclear transport factors, importin α, importin β, and Ran, between the nucleus and the cytoplasm. The cytoplasmic distribution of importin α and β, the nucleoplasmic localization of Ran in non-apoptotic interphase cells are converted to nuclear importins and cytoplasmic Ran in apoptotic cells (Ferrando-May et al., 2001; Ferrando-May, 2005; Wilde and Zheng, 2009). CPV-induced DNA damage modulates cell cycle progression and induces apoptosis (Luo and Qiu, 2013; Zhao et al., 2016). Apoptosis is known as the major cell death pathway at the final stages of CPV infection (Gupta et al., 2016; Nykky et al., 2010), however, its role in the nuclear egress of capsids remains to be determined.

**FIGURE 7:**
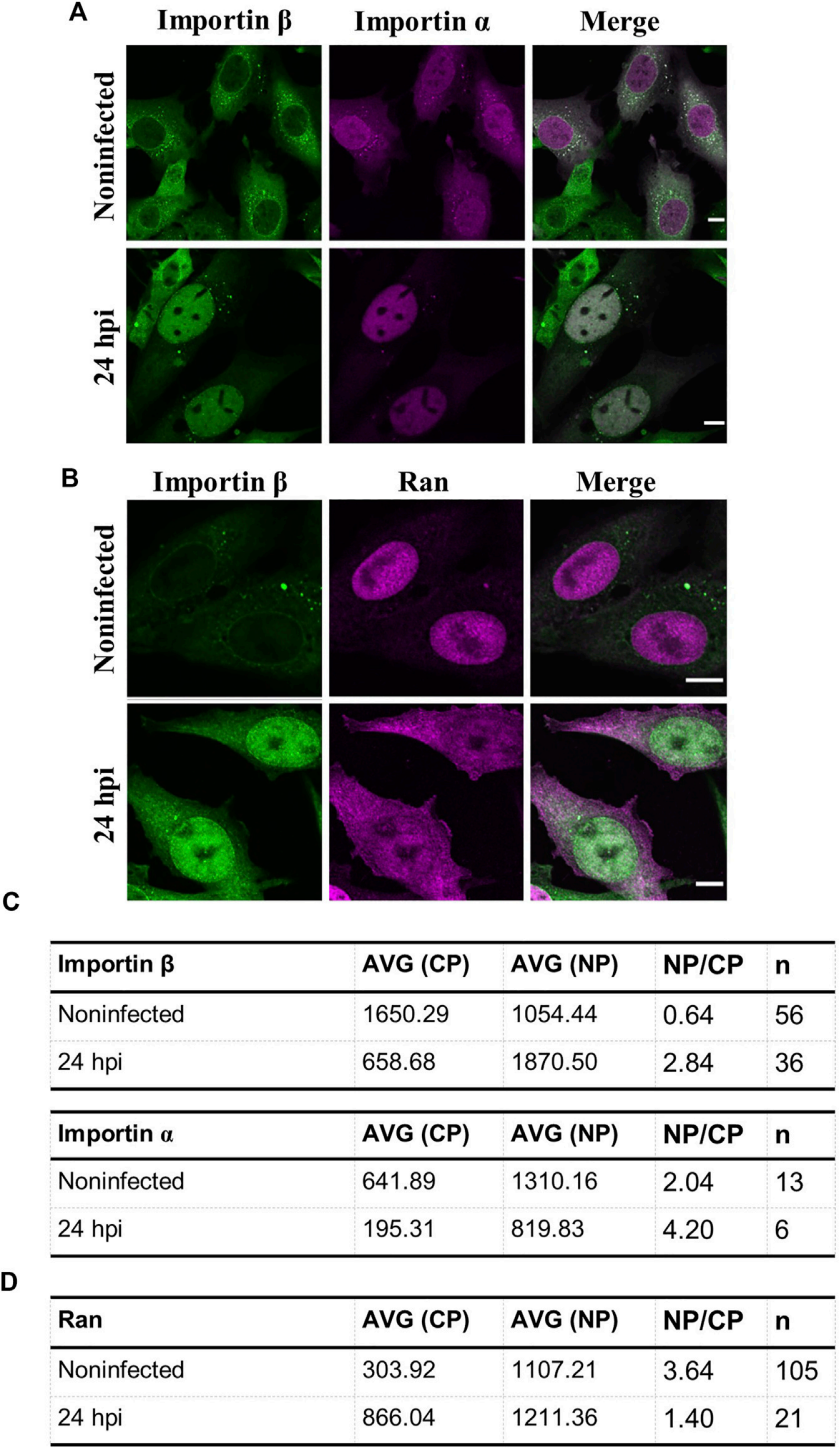
Intracellular distributions of importins and ran are changed in infected samples. Representative confocal images of intracellular distribution of **(A)** importin β together with either importin α or **(B)** Ran in non-infected and infected NLFK cells at 24 hpi. The cells were immunolabeled with antibodies against importin β (green) and ran (magenta). Importin a was expressed as fluorescent importin α-EGFP (magenta). Scale bars, 10 µm **(C)** Quantitative analysis showing the nucleus-to-cytoplasmic ratio of importin β, α, and **(D)** Ran in non-infected and infected cells. Nucleus-to-cytoplasmic ratios were calculated from averaged intensities of nuclear transport factors in the nucleus and the cytoplasm. Nucleus-to-cytoplasmic ratio changes of all the studied nuclear transport factors were statistically significant *p* < 0.05, and statistical analysis was performed by using Student´s *t*-test.

As a verification of CPV-induced apoptosis, we analyzed the nuclear distribution of transport factors. Nuclear localization of importin β was used as an infection marker since visualization of infected HeLa cells demonstrated that both importin β and the viral capsid protein VP2 were accumulated into the nucleus at 24 hpi. In contrast, importin β showed mostly perinuclear and cytoplasmic localization in non-infected cells (Fig S4). Moreover, importin α (importin-α-GFP) and importin β was accumulated in the nucleus at 24 hpi in NLFK cells (Figure 7A). Ran was localized both to the nucleus and to the cytoplasm in infected cells, in contrast to the exclusively nuclear localization of Ran in non-infected cells (Figure 7B). Quantitative analyses of nucleus-to-cytoplasmic ratios in infected and non-infected cells confirmed the nuclear accumulation of importins and cytoplasmic localization of Ran in infected cells. The nucleus-to-cytoplasmic ratio for importin α in infected and non-infected cells were 4.20 and 2.04, and for importin β 2.84 and 0.64 (Figure 7C). At the same time, the amount of cytoplasmic Ran was increased with ratios of 3.64 in non-infected cells in comparison to 1.40 in infected samples (Figure 7D).

Taken together, late infection is characterized by an apoptotic alteration of nucleocytoplasmic localization of nuclear transporters, importins, and Ran, suggesting that the apoptotic events are induced in infected cells.

### The nuclear exit of capsids is decreased by caspase 3 inhibition

Finally, to study the effects of G2/M checkpoint-related apoptotic events for nuclear egress of capsids, we applied inhibition of caspases in late-infection. Caspases are cysteine-dependent proteases that are actively involved in the execution of apoptosis (McIlwain et al., 2013). One of the downstream caspases, caspase 3, is activated in early apoptosis (Varghese et al., 2003). It is essential for apoptotic events such as the formation of apoptotic bodies, chromatin condensation, and DNA fragmentation (Jänicke et al., 1998; Woo et al., 1998). Caspase 3 has also a role in the regulation of mitosis (Swe and Sit, 2000; Hsu et al., 2006; Hashimoto et al., 2011; Lee et al., 2011). Notably, caspase 3, which is also involved in the induction of NE breakage, has a role in the nuclear import of MVM (Cohen et al., 2011).

**FIGURE 8:**
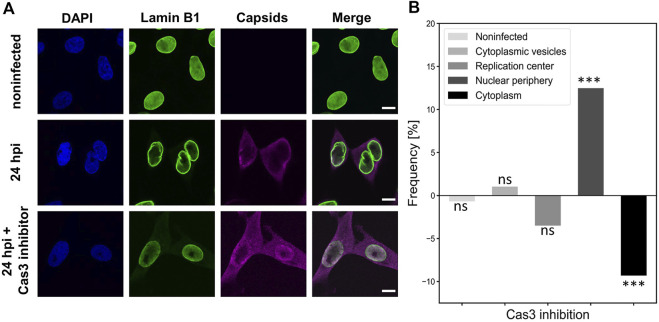
Caspase 3 inhibition leads to decreased nuclear egress of capsids **(A)** Non-infected and infected NLFK cells in the absence and the presence of caspase 3 inhibitor at 24 hpi. The cells were immunolabeled with antibodies against lamin B1 (green), intact capsids (magenta) and blue corresponds to DAPI staining. Scale bars, 25 µm. **(B)** Intracellular localization of capsids in non-treated and caspase 3 treated infected cells. Five capsid localization classes described in Figure 6A are shown. The horizontal line at value 0 shows capsid distribution in untreated infected cells, and positive and negative values represent inhibitor-induced increase and decrease in the specific localization of capsids. Significant differences tested with the chi-square test are denoted as *** (*p* < 0.001), and non-significant as ns (*p* > 0.05).

To characterize the importance of early apoptosis for the nuclear exit of viral capsids, we treated infected cells with a caspase 3 inhibitor (Z-DEVD-FMK), which is a peptide that prevents the induction of early apoptosis (Garcia-Calvo et al., 1998). Figure 8A shows that the amount of cytoplasmic capsid label was visibly decreased in the presence of caspase 3 inhibitor in comparison to untreated NLFK cells at 24 hpi. The image data analysis using capsid localization classes described above (Figure 6C) confirmed that the nuclear egress of capsids was significantly decreased during caspase 3 inhibition. Simultaneously, capsid retention close to the NE was increased. Interestingly, the early steps of infection shown by capsid localization into cytoplasmic vesicles were reduced in the presence of the inhibitor (Figure 8B). This implies that the inhibition of early apoptosis or mitosis does not only affect the nuclear egress of capsid but might also have an influence on capsid entry.

Together, these results suggest that caspase 3 and early apoptosis have a potential role in enhancing the nuclear egress of viral capsids. However, it remains possible that caspase 3 might also affect the mitotic facilitation of capsid egress.

### The infection leads to epigenetic activation of host genes involved in mitosis and apoptosis

Our findings suggested that CPV egress is enhanced by the promotion of G2/M checkpoint activation and regulation of apoptosis. To follow up on these observations and to analyze specific virus-induced changes in the epigenetic landscape of cell cycle and death, we surveyed the histone 3 lysine 27 acetylation (H3K27ac) by ChIP–seq. H3K27ac marks enhancer*-*specific modifications required for the activation of target gene transcription (Heintzman et al., 2009, 2007; Wang et al., 2008).

**FIGURE 9:**
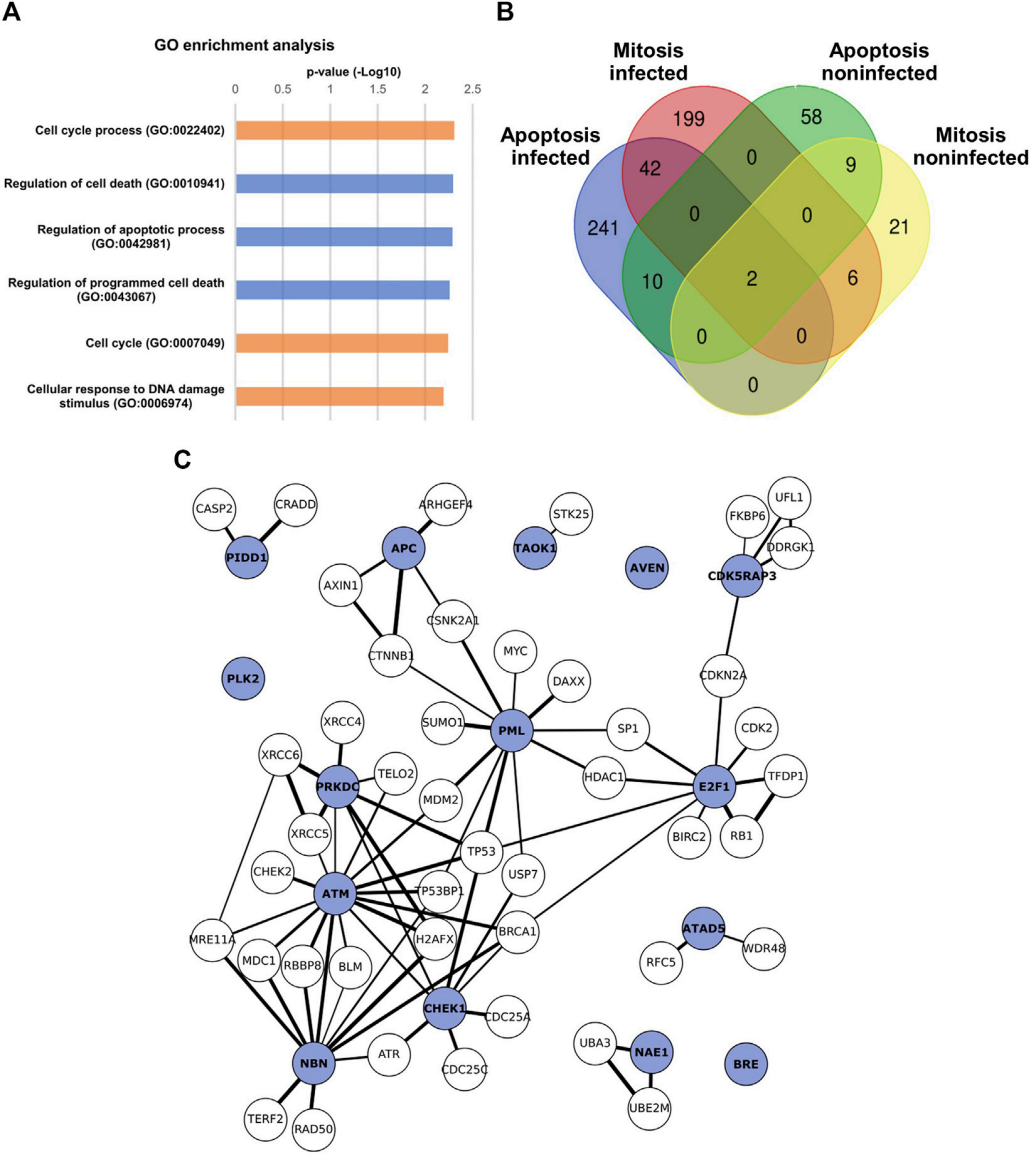
Infection leads to H3K27 acetylation and deacetylation of host genes associated with mitotic and apoptotic events ChIP-seq analysis of H3K27ac acetylation in non-infected and infected NLFK cells at 24 hpi. **(A)** Cell cycle and apoptosis-related GO terms in infected cells detected by H3K27Ac enrichment analysis. Bar charts of the *p*-*v*alues (-log10) for the differentially acetylated GO categories with acetylation loss (blue) and acetylation gain (orange) between non-infected and infected samples. False discovery rate <0.05 was used as a threshold to enroll significantly enriched GO categories. **(B)** A Venn diagram representing the number of genes with H3K27ac enrichment in regions surrounding the transcription start sites associated with mitosis and apoptosis, and their overlap in infected and non-infected cells. Genes related to mitosis were pooled from GO classes: regulation of mitotic cell cycle (GO:0007346), mitotic cell cycle (GO:0000278), mitotic cell cycle process (GO:1903047), and regulation of mitotic cell cycle phase transition (GO:1901990). Genes involved in apoptosis were negative in the regulation of the apoptotic process (GO:0043066), positive in the apoptotic process (GO:0043065), apoptotic process (GO:0006915), and apoptosis (GO:0042981). **(C)** Network summarizing a list of enriched GO terms proteins in late infection show proteins as circles arranged based on their interactions. The protein-protein interactions originate from STRING (https://string-db.org/). The blue color represents the enriched proteins while the white color represents the first-order interacting protein. The thicknesses of lines are proportional to the interaction confidence.

Analysis of the host genome indicated that the H3K27ac signal was increased in infection (24 hpi) in comparison to non-infected NLFK cells. In total, the H3K27ac profile contained 43512 peaks in control cells compared to 54174 in CPV-infected cells (24% increase after infection). Gene Ontology (GO) enrichment analysis of the H3K27ac ChIP-seq demonstrated either gain or loss of acetylation in mitotic and apoptotic categories in comparison to non-infected cells (Figure 8A). Significantly enriched categories with H2K27ac loss were regulation of cell death (GO:0010941), regulation of apoptotic process (GO:004281), and regulation of programmed cell death (GO:0043067), whereas GO categories significantly enriched with acetylation gain were cell cycle process (GO:0022402), cell cycle (GO:0007049) and cellular response to DNA damage stimulus (GO:0006974) (Table S1). Further analyses of acetylation in infected cells showed that H3K27ac was enriched significantly in genes related to mitosis signaling pathways including several GO classes such as regulation of mitotic cell cycle (GO:0007346), mitotic cell cycle (GO:0000278), mitotic cell cycle process (GO:1903047), and regulation of mitotic cell cycle phase transition (GO:1901990) in infected samples. The infection led also to a significant increase in acetylation of transcription start sites region related to apoptosis. These signaling pathways pooled from GO categories were negative regulation of apoptotic process (GO:0043066), positive regulation of apoptotic process (GO:0043065), apoptotic process (GO:0006915), and apoptosis (GO:0042981). In infected cells, the regions with high H3K27ac included 205 mitotic and 251 apoptotic genes and 44 genes which belonged to both categories, whereas in non-infected cells 27 mitotic, 68 apoptotic, and 11 shared genes were acetylated. Moreover, six mitotic, 10 apoptotic, and two shared genes were activated both in the infected and non-infected cells (Figure 9B). Venn´s analysis of ChIPseq-enriched activated genes unique to the late infection indicated a total of 42 genes associated with apoptotic and mitosis signaling (Figure 9B). These 42 genes were analyzed further by using STRING (https://string-db.org/) online analysis tool for identifying protein-protein interaction networks. Next, gene ontology (GO) enrichment analysis for biological processes (BP) was performed. The BP enrichment analysis revealed enrichment of e.g., cell cycle checkpoint, signal transduction involved in G2/M transition checkpoint, signal transduction involved in cell cycle checkpoint, G1/S checkpoint, G1/S transition checkpoint, G2/M checkpoint, G2/M transition checkpoint (GO:0000075); processes participating mitotic cell cycle (GO:1903047); DNA integrity checkpoint signaling (GO:0031570); DNA damage checkpoint signaling (GO:0000077); negative regulation of cell cycle G2/M phase transition (GO:1902750); regulation of cell cycle G2/M phase transition (GO:1902749); and signal transduction in response to DNA damage (GO:0042770). In addition, mitotic events such as mitotic DNA integrity checkpoint signaling (GO:0044774), mitotic DNA damage checkpoint signaling (GO:0044773), intrinsic apoptotic signaling pathway in response to DNA damage (GO:0008630), negative regulation of G2/M transition of the mitotic cell cycle (GO:0010972) were enriched. The analysis had a false discovery rate i.e., *p*-value *p* < 0.01 corrected for multiple testing within each category using the Benjamini–Hochberg procedure (Table 2).

From the associated GO terms, the top 15 genes that appeared to be involved in most of these pathways were recognized (Figure 9C, Table S 2, and Table S 2). Interestingly, among these was the Chk1/Chk2(Cds1) pathway, which is known to be activated in response to DNA damage resulting in the conversion and/or maintenance of cyclin B1:Cdk1(Cdc2) complex in its Tyrosine 15 phosphorylated (inactive) state. Activation of Chk1 can be done *via* ATM, which was also included in the analysis results. Specifically, the dsDNA breaks are considered to be processed in an ATM-dependent manner causing primarily ATR and hence Chk1 activation. Based on our findings, it is thus tempting to speculate that the DNA damage-induced G2/M checkpoint activation in CPV infection might occur *via* ATM-dependent cyclin B1/Cdk1 inhibition in a Chk1-mediated manner. However, as signaling pathways were not in the scope of this study, this needs to be further investigated in the future.

Taken together, these results support the findings that DNA-damage-induced G2/M checkpoint regulation takes place in late infection. Our ChIP-seq findings are consistent with the model that mitosis and apoptosis are regulated at late infection. Both of these pathways lead to NE leakiness, thereby potentially contributing to the enhancement of the nuclear egress of viral capsids.

## Discussion

**FIGURE 10:**
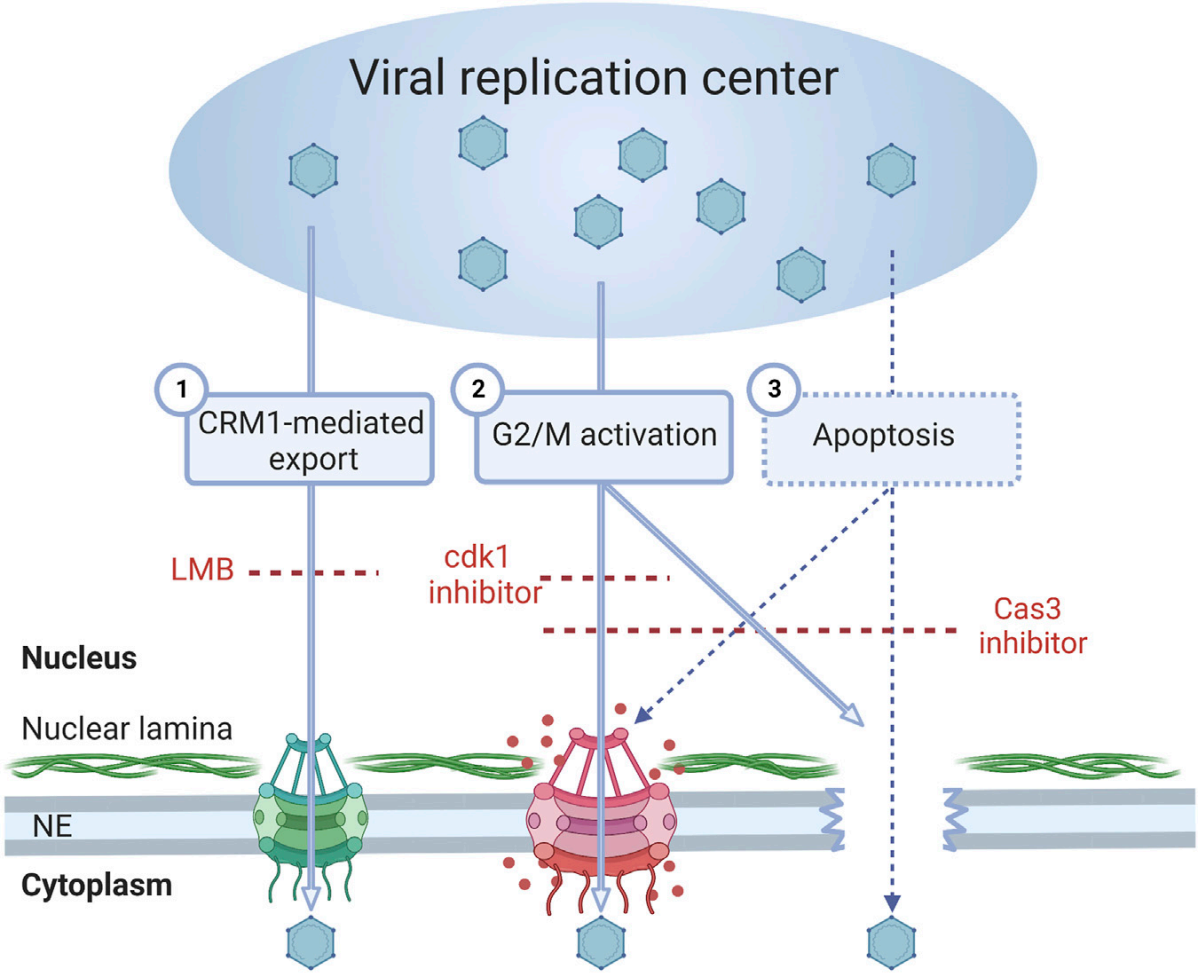
Alternative modes of nuclear exit of parvoviruses. Parvovirus capsids are assembled and matured in the nucleus of the host cells. During the progression of infection, the virus-induced damage response is followed by the pre-mitotic cell cycle arrest. (1) Earlier studies have shown that MVM capsids are released from the nucleus to the cytoplasm *via* an export factor CRM1-mediated nuclear transport pathway through nuclear pore complexes. CRM1*-*dependent nuclear export is inhibited by leptomycin B (LMB). Our studies suggest that two additional nuclear exit pathways may play a role in capsid egress. (2) First, we showed that infection induces activation G2/M checkpoint. Upon activation of mitosis, the nuclear lamina undergoes disintegration, which is followed by nuclear envelope breakage, thereby allowing passive diffusion of capsids through the nuclear envelope. Inhibition of cyclin-dependent kinase 1 (cdk1) blocks the nuclear envelope leakage. (3) Second, infection induces caspase-dependent apoptosis in infected cells. During the early stages of the apoptosis, the capsid egress is facilitated by an increase in the envelope permeability caused by the disintegration of the nuclear lamina, together with the disassembly and enlargement of NPCs. Caspase 3 also has a potential role in the regulation of mitosis. The apoptosis-induced nuclear envelope leakiness can be prevented by exposure to a Caspase 3 (Cas3) inhibitor.

The egress of parvovirus capsids through the nuclear pores, as previously described for MVM, is an active process mediated by nuclear export receptor CRM1, which interacts with the viral NS2 protein (Eichwald et al., 2002; Engelsma et al., 2008). The alternative model, suggested by the present work, is that the nuclear egress of CPV capsids is facilitated by the regulation of G2/M checkpoint and apoptosis, which both induce an increase in NE permeability (Figure 10).

In parvovirus infection, the viral gene expression leads to the synthesis of viral capsid proteins and the formation of capsid subunits, phosphorylated VPs trimers (Lombardo et al., 2000; Riolobos et al., 2006; Cotmore and Tattersall, 2013). Upon progression, to the early S phase, the capsid subunits are transported into the nucleus where they form empty capsids. Maturation into DNA-filled capsids occurs when cells are arrested at the late S/G2 phase (Gil-Ranedo et al., 2015). After assembly, the capsids must translocate into the cytoplasm. Our results suggest that in addition to the previously described CRM1-mediated nuclear egress of capsids, alternative routes of exit are used. One of these pathways depends on the regulation of G2/M checkpoint, which induces mitotic disintegration of the NE, leading to increased nuclear permeability (Feldherr and Akin, 1990; Dultz et al., 2009). This is consistent with the notion that in non-infected cells the early mitotic disassembly of nuclear pore complexes is followed by the phosphorylation and disintegration of the nuclear lamina, and the NE breakdown (Goss et al., 1994; Thompson and Fields, 1996; Laurell et al., 2011; Laurell and Kutay, 2011; Linder et al., 2017). When the G2/M checkpoint is activated the cell cycle proceeds towards mitosis, which is driven by the activation of cyclin B1–Cdk1 as the cyclin subunit is phosphorylated in the cytoplasm (Li et al., 1997; Hagting et al., 1999; Jackman et al., 2003; Lindqvist et al., 2009; Gavet and Pines, 2010). The nuclear translocation of activated cyclin B1 is known to mark a restriction point for the transition from G2 to the mitotic phase (Dulić et al., 1998; Rhind and Russell, 2012). Moreover, in cells with DNA damage nuclear cyclin B1 has a role in the regulation of apoptosis (Porter et al., 2003). The nuclear transport of Cdk1-cyclin B1 is also accompanied by the NE breakdown (Gong et al., 2007). It has been reported that in parvovirus-infected cells, where signals induced by DNA damage prevent G2/M transition, the activation and nuclear accumulation of Cdk1-cyclin B1 is inhibited (Deming et al., 2001; Charrier-Savournin et al., 2004; Adeyemi and Pintel, 2014, 2012). The viral NS1 protein plays multiple roles in these processes, including induction of DNA breaks (Adeyemi et al., 2010; Hristov et al., 2010; op de Beeck and Caillet-Fauquet, 1997) and regulation of the cell cycle (Morita et al., 2003; op de Beeck et al., 2001; op de Beeck and Caillet-Fauquet, 1997). Our studies showed that one-third of infected cells at 24 hpi presented nuclear accumulation of cyclin B1. Inhibition of cell cycle progression towards the M phase through the inactivation of Cdk1 led to a decrease in the portion of cytoplasmic capsids, suggesting that the G2/M checkpoint activation has a potential role in the nuclear egress of capsids. The G2/M checkpoint regulation in infection is also supported by our findings that genes playing a role in mitosis are activated. As described earlier, parvovirus-induced mitotic disintegration of the NE in early infection is connected to the nuclear entry of viral capsids (Porwal et al., 2013). Our results suggest that at the late stages of infection activation of mitosis may also play a role in the nuclear egress of progeny capsids.

Parvoviruses induce host DNA disintegration, DDR activation, and apoptosis (Roos and Kaina, 2006; Harper and Elledge, 2007; Adeyemi et al., 2010; Luo and Qiu, 2013). The consequences of DDR in cells depend on the severity of DNA damage (Surova and Zhivotovsky, 2013). Mild damage, such as single-stranded DNA breaks, leads to the induction of cell-cycle arrest (Bantele et al., 2019), whereas more detrimental and irreparable injury leads to the induction of both cell-cycle arrest and cell death programs, such as apoptosis (Norbury and Zhivotovsky, 2004; Surova and Zhivotovsky, 2013; Feringa et al., 2018) (1. In addition, prolonged mitotic arrest leads to the activation of apoptosis (Orth et al., 2012). Studies with adenovirus demonstrated that virus-induced G2/M arrest was followed by apoptosis (Brand et al., 1999). In apoptotic cells, the caspase-dependent disassembly of nuclear pores and the nuclear lamina increases the NE permeability (Kihlmark et al., 2004, 2001; Roehrig et al., 2003; Ferrando-May, 2005; Strasser et al., 2012; Shahin, 2017; Lindenboim et al., 2020). Our studies suggest that virus-induced apoptosis, which leads to enhanced nucleocytoplasmic diffusion, might be used as an alternative egress pathway for viral capsids. Our findings also demonstrate nucleocytoplasmic conversion of nuclear transport factors, importins, and Ran, characteristic of apoptotic cells (Ferrando-May et al., 2001; Ferrando-May, 2005). The ran gradient is also disrupted during DNA damage response (Dworak et al., 2019), and importin α is accumulated into the nucleus in response to cellular stress (Miyamoto et al., 2004). The role of apoptosis in capsid egress is supported by the observed caspase 3 inhibition-induced decrease in the nuclear exit of capsids. However, although the role of caspase 3 in mitosis has remained controversial (Swe and Sit, 2000; Hsu et al., 2006; Hashimoto et al., 2011; Lee et al., 2011), we cannot rule out that caspase 3 inhibition might affect both apoptotic and mitotic disintegration of the NE and capsid exit. It is also important to note that the host genes involved in apoptosis are regulated during CPV infection. Altogether, while it is plausible that the nuclear export of CPV capsids is enhanced by CRM1-mediated export and early mitosis-induced disassembly of the nuclear lamina, it seems possible that apoptosis-induced disruption of the NE and nuclear pore integrity promotes the nuclear egress of parvoviral capsids.

Parvovirus infection is characterized by viral capsid egress through the NE to the cytoplasm at the late stages of infection. While it is known that parvoviral capsids use active CRM1-mediated nuclear export through the nuclear pore complex, our studies suggest that changes in nuclear permeability during regulation of G2/M checkpoint and apoptosis may play a significant role in the nuclear egress of progeny parvoviruses.

## Data Availability

The datasets presented in this study can be found in online repositories. The names of the repository/repositories and accession number(s) can be found below: https://www.ncbi.nlm.nih.gov/, GSE77785.
