# Supplementary material for: G2/M checkpoint regulation and apoptosis facilitate the nuclear egress of parvoviral capsids
Source: Front Cell Dev Biol. 2022 Dec 8;10:1070599. doi: 10.3389/fcell.2022.1070599 (PMC9773396; doi:10.3389/fcell.2022.1070599)
Supplement: Supplementary file 2 [file Table1.DOCX]

**G2/M checkpoint regulation and apoptosis facilitate the nuclear egress of parvoviral capsids**

Salla Mattola^1*^, Elina Mäntylä^2*^, Vesa Aho^1^, Sami Salminen^1^, Mikko Oittinen^3^, Kari Salokas^4^, Jani Järvensivu^1^, Satu Hakanen^1^, Teemu O Ihalainen^2^, Keijo Viiri^3^, and Maija Vihinen-Ranta^2#^

Supplementary Material

# Supplementary Figures and Table

## Supplementary Figures


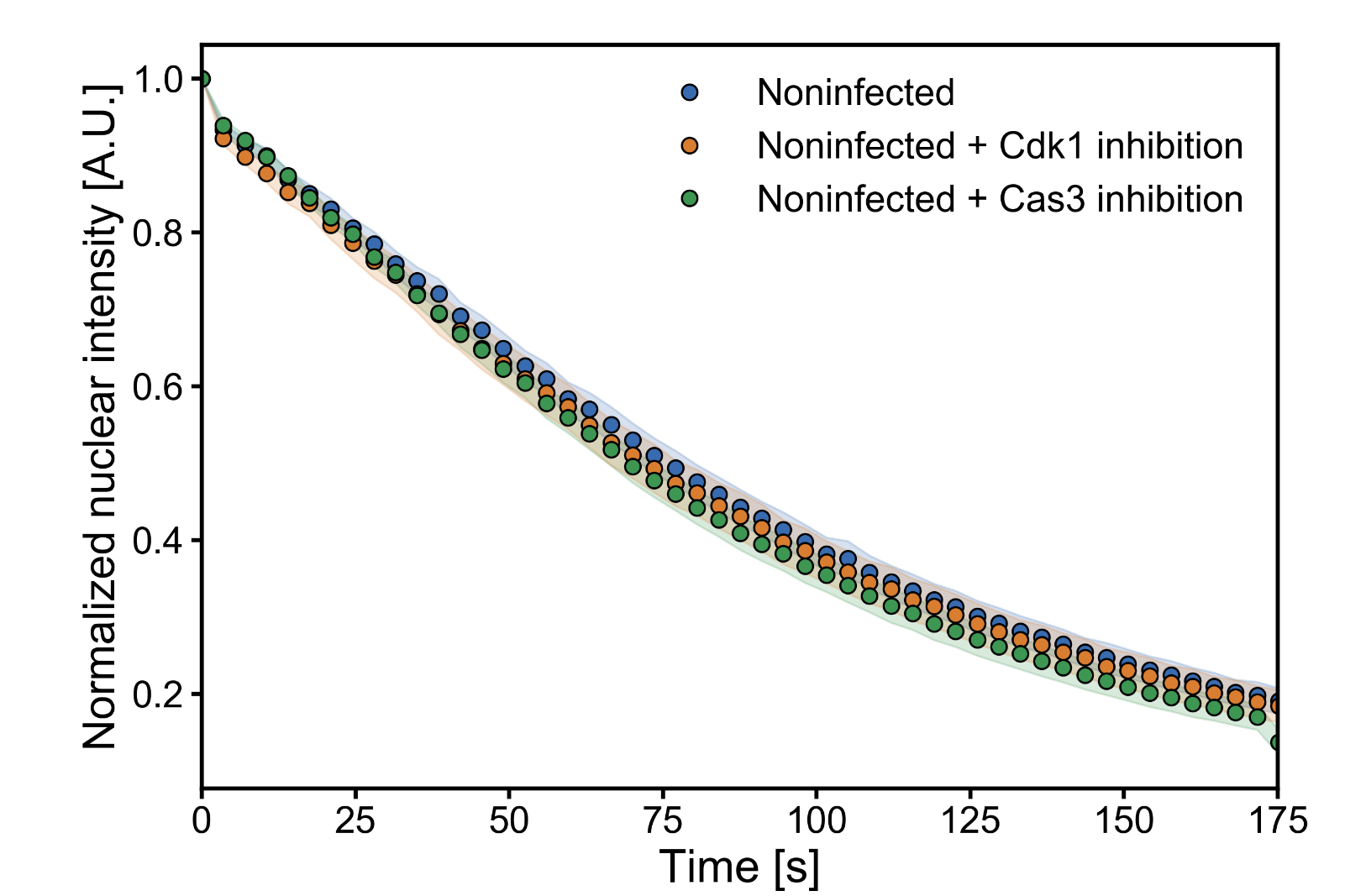


**Figure S1. The nuclear envelope permeability of noninfected cells treated with Cdk1 and caspase inhibitors**

FLIP analyses of nuclear envelope permeability in noninfected NLFK cells in the presence and absence of inhibitors of Cdk1 (RO-3306) and apoptosis (caspase 3 inhibitor). Fluorescence loss was quantified during a period of 175 s in a nucleoplasmic region during continual photobleaching of the cytoplasm. The fluorescence loss curves show the relative fluorescence intensity of noninfected untreated control cells (blue) (n=12), noninfected control cells in the presence of cdk1 inhibitor, RO-3306 (orange) (n=12), and caspase 3 inhibitor (green) (n=14). The shaded areas around the data points represent the standard error of the mean.


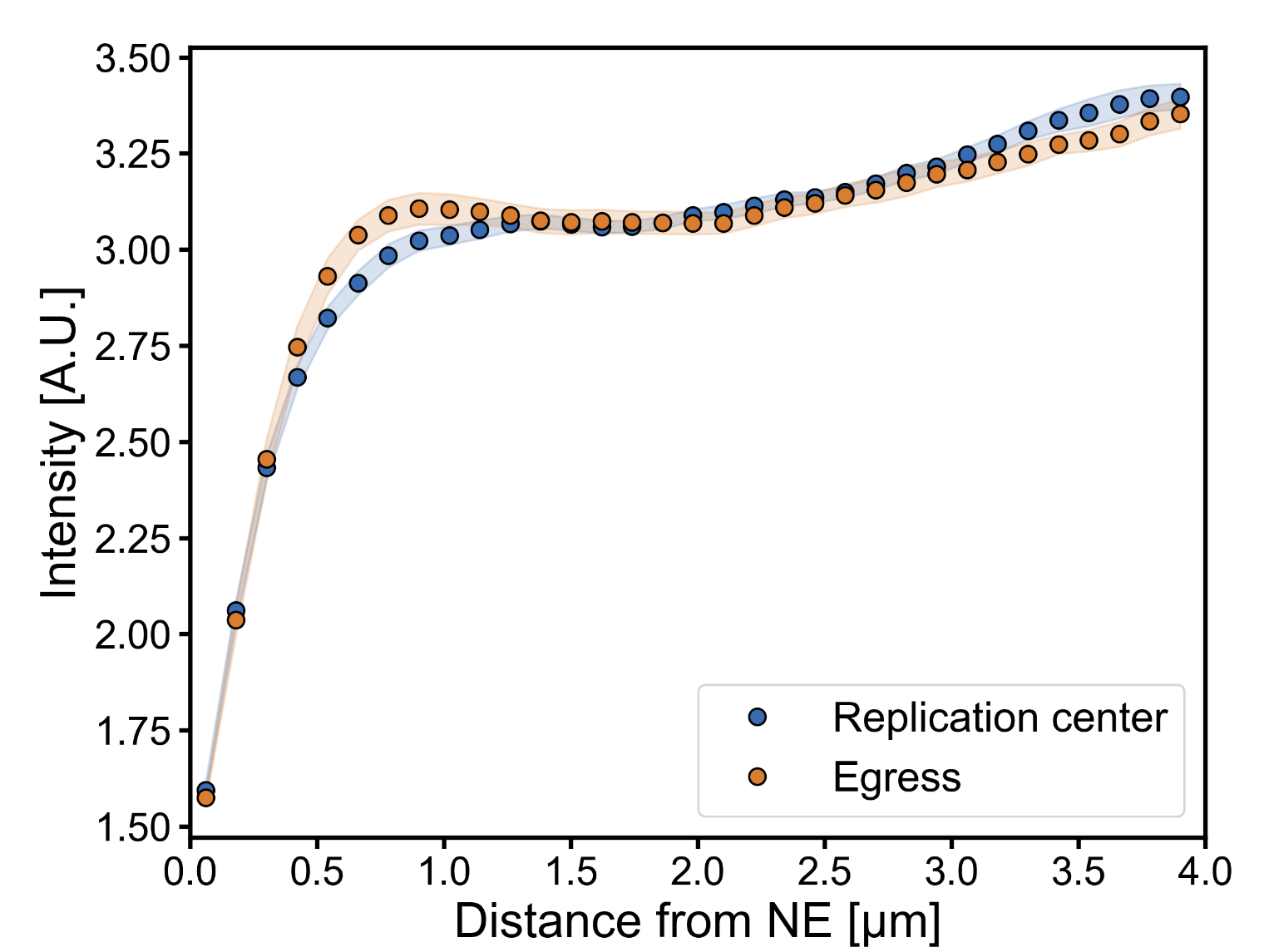
**Figure S2. Chromatin distribution in infected cells**

Nuclear distribution of NucBlue-labeled chromatin as a function of the distance from the nuclear envelope in infected Hela cells at 24 hpi. Infected cells are divided into replication center class with capsids accumulated into the central nuclear region, and egress class with capsids localized both to the nuclear periphery and in the cytoplasm. The shaded areas around the data points represent the standard error of the mean (n=10).


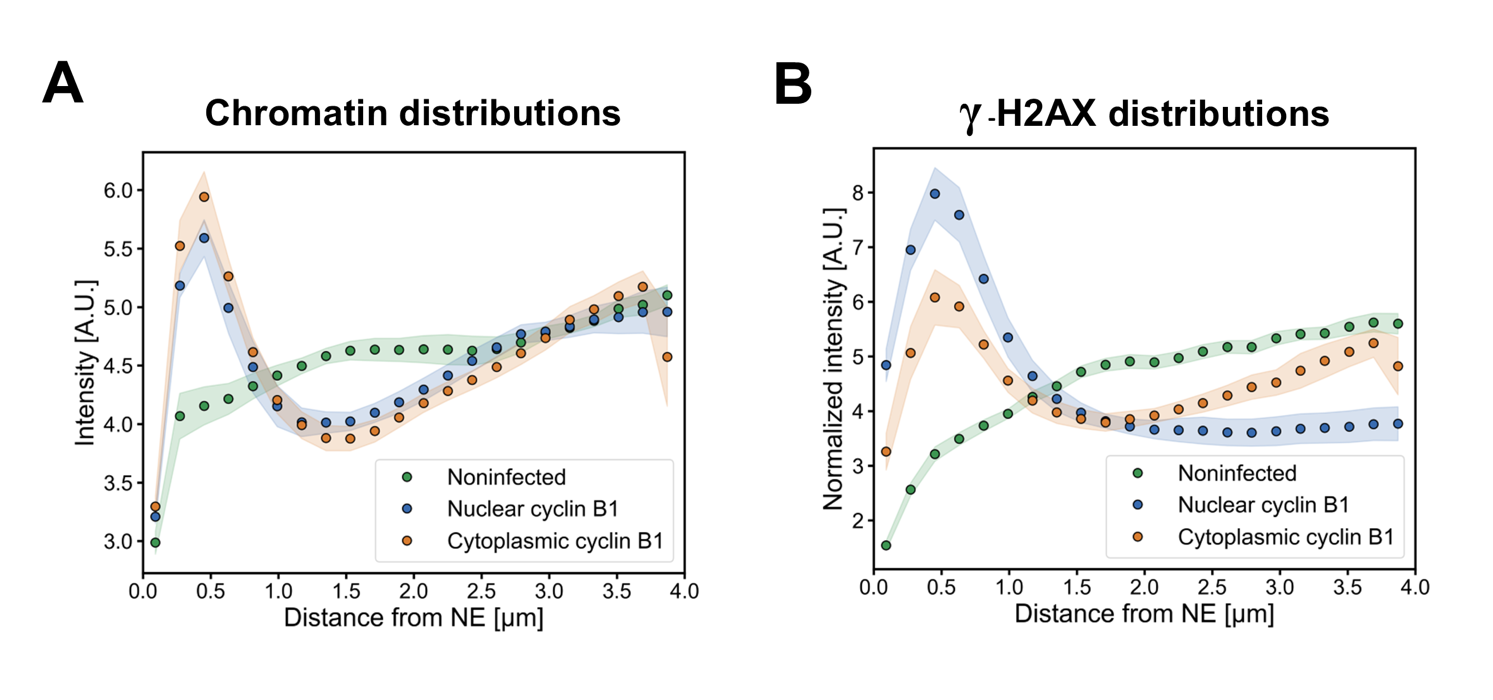


**Figure S3. Nuclear distributions of chromatin and γ-H2AX in noninfected and infected cells with either nuclear or cytoplasmic cyclin B1 localization** Analyses of the distributions of (**A**) chromatin and (**B**) γ-H2AX as a function of the distance from the nuclear envelope in infected (24 hpi) cells with nuclear (n=9) (blue) and cytoplasmic (n=17) (orange) cyclin B1, and in noninfected (n=10) (green) NLFK cells. The shaded areas around the data points represent the standard error of the mean.


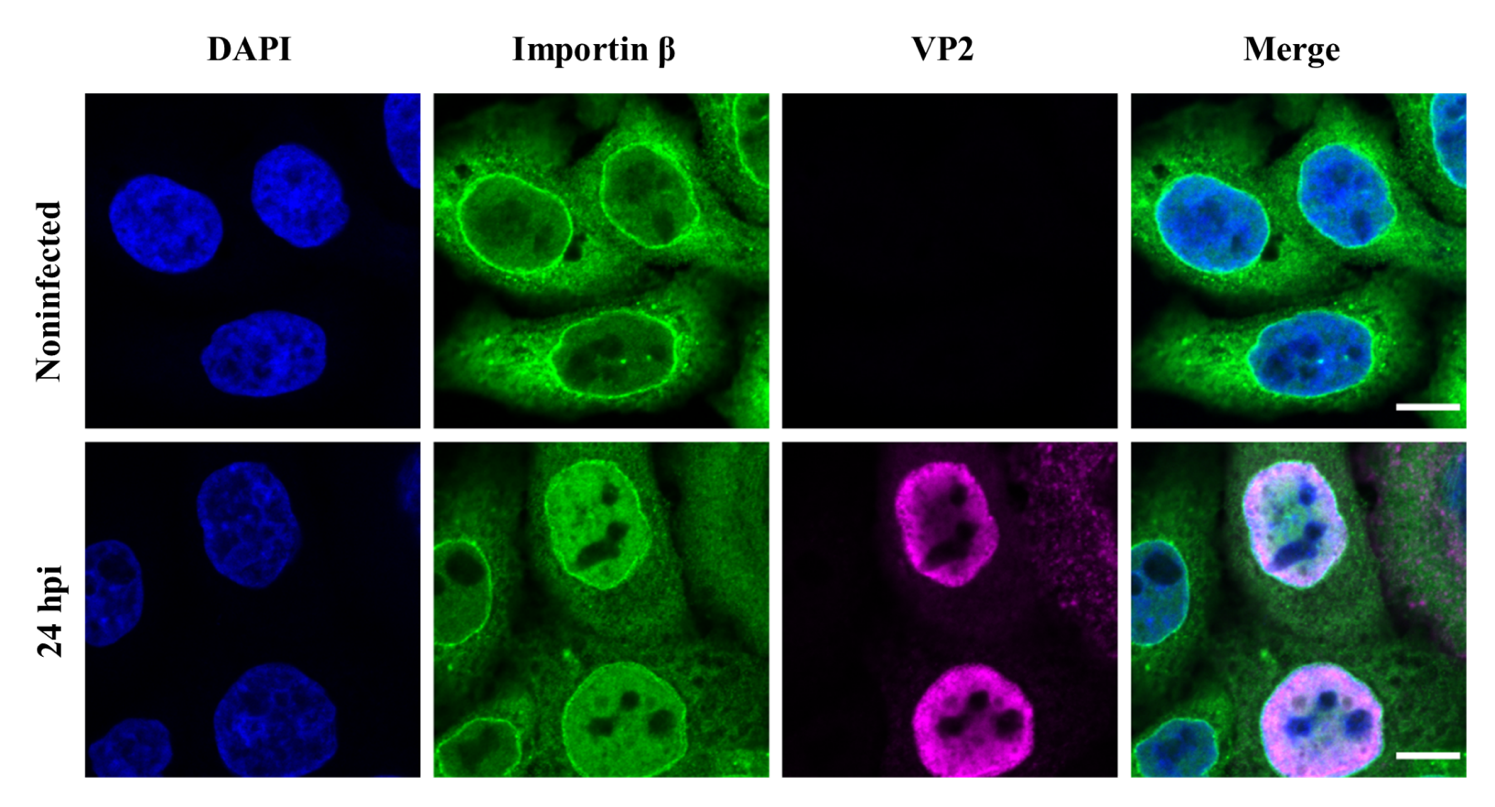


**Figure S4. Intracellular distributions of importin β in noninfected and infected cells**

Representative confocal images of intracellular distribution of importin β in noninfected and infected Hela cells at 24 hpi. Cells were immunolabeled with antibodies against importin b (green) and VP2 (magenta). Blue corresponds to DAPI staining. Scale bars, 10 µm.

## Supplementary Tables


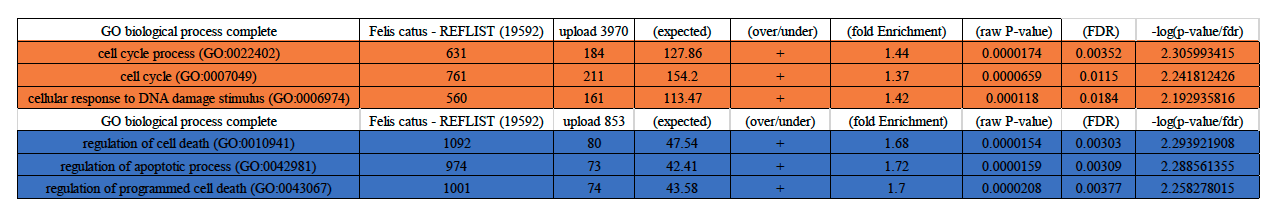


**Table S1. Cell cycle and apoptosis related GO terms were enriched in biological process analyses**

GO categories enriched within acetylation gain (orange) and acetylation loss (blue) analyses of infected cells compared to noninfected cells. False discovery rate <0.05 was used as a threshold to enrol significantly enriched GO categories. Statistical significances were determined with Fisher's Exact test.

**Table S2. Enriched activated genes in late infection**

The table presents ChIPseq-enriched activated genes and their roles in biological processes in CPV-infected NLFK cells at 24 hpi. The most important 15 genes that are involved in several biological pathways in the cell cycle and death are shown in blue.

The Table is provided as a separate excel file
